# Metabolomic Profile of Personalized Donor Human Milk

**DOI:** 10.3390/molecules25245783

**Published:** 2020-12-08

**Authors:** Monica F. Torrez Lamberti, Evon DeBose-Scarlett, Timothy Garret, Leslie Ann Parker, Josef Neu, Graciela L. Lorca

**Affiliations:** 1 Department of Microbiology and Cell Science, Genetics Institute, Institute of Food and Agricultural Sciences, University of Florida, Gainesville, FL 32611, USA; m.torrezlamberti@ufl.edu (M.F.T.L.); escarlett@ufl.edu (E.D.-S.); 2 Department of Pathology, Immunology, and Laboratory Medicine, College of Medicine, University of Florida, Gainesville, FL 32611, USA; tgarrett@ufl.edu; 3 College of Nursing, University of Florida, Gainesville, FL 32611, USA; parkela@ufl.edu; 4 Department of Pediatrics, Division of Neonatology, University of Florida, Gainesville, FL 32611, USA; neuj@peds.ufl.edu

**Keywords:** mother’s own milk, microbiota, metabolomics

## Abstract

Human milk could be considered an active and complex mixture of beneficial bacteria and bioactive compounds. Since pasteurization drastically reduces the microbial content, we recently demonstrated that pasteurized donor human milk (DHM) could be inoculated with different percentages (10% and 30%) of mother’s own milk (MOM) to restore the unique live microbiota, resulting in personalized milk (RM10 and RM30, respectively). Pasteurization affects not only the survival of the microbiota but also the concentration of proteins and metabolites, in this study, we performed a comparative metabolomic analysis of the RM10, RM30, MOM and DHM samples to evaluate the impact of microbial restoration on metabolite profiles, where metabolite profiles clustered into four well-defined groups. Comparative analyses of DHM and MOM metabolomes determined that over one thousand features were significantly different. In addition, significant changes in the metabolite concentrations were observed in MOM and RM30 samples after four hours of incubation, while the concentration of metabolites in DHM remained constant, indicating that these changes are related to the microbial expansion. In summary, our analyses indicate that the metabolite profiles of DHM are significantly different from that of MOM, and the profile of MOM may be partially restored in DHM through microbial expansion.

## 1. Introduction

For years, the practice of formula feeding babies was highly prevalent, supported by studies which reported that formula-fed infants gain more weight than breastfed infants [1,2]. However, mother’s own milk (MOM) provides infants with a greater variety of bioactive components (i.e., nutritional and immune mediators) than just those related to weight gain [3,4]. In light of this, the World Health Organization recommends exclusive breastfeeding during the first six months of life [5] highlighting the importance of MOM in the initial stages of infants’ physical and cognitive development [6,7]. Several reports have shown lower incidence of necrotizing enterocolitis (NEC) and other infections in breastfed infants [8,9,10]. It has been reported that bovine-based products negatively impact the integrity of the gut by increasing intestinal permeability [11]. In addition, formula-fed infants are more likely to be colonized by gram-negative bacteria, leading to an up-regulated inflammatory response [12].

Improved cognitive development and decreased incidence of obesity, diabetes, and cardiovascular disease were also reported [8,9,10] with MOM feedings when compared to bovine milk–based products [13,14,15,16]. These outcomes were also positively correlated with better feeding tolerance in very-low-birth-weight infants (<1500 g) [17]. In the absence of sufficient amounts of MOM, the American Academy of Pediatrics recommends feeding preterm infants with pasteurized donor human milk (DHM) instead of formula [18].

DHM is pasteurized to decrease the presence of potential pathogens; however, the levels of beneficial microorganisms, hormones, human milk oligosaccharides (HMOs), and other immunological factors are also affected [19,20,21,22,23]. These changes in composition are correlated with decreased efficacy in preventing NEC and late-onset sepsis [10,14,24,25,26]. Numerous compounds such as myoinositol [27], growth factors [28,29], and antioxidants [30] that are thought to contribute to improving infants’ health, are considerably reduced or even absent in DHM. In addition, lactoferrin levels are reduced by around 50% after freezing MOM [31]. Pasteurization further reduces the concentration of this compound by up to 88% [32]. Additionally, Bullen (1972) reported that the milk fortification process further reduces the remaining bioactivity of lactoferrin due to the increase in iron content [33]. Despite efforts to improve the pasteurization processes to minimize the impact on milk composition, there is still a sizable difference between some MOM and DHM components.

The human milk metabolome was investigated using proton nuclear magnetic resonance spectroscopy, as well as liquid chromatography coupled with mass spectrometry [34,35]. The use of these approaches allowed the identification of unique metabolite profiles in human milk when compared to bovine milk or formula [36]. Similarly, extensive lipidomic analysis also highlighted these differences [36]. Previous studies have also determined that a myriad of factors can alter the metabolite profile of MOM, including genetic factors, ethnicity, lactation phase, gestational age at birth, diet, HMO secretor status and mother disorders such as gestational diabetes and preeclampsia [37,38,39,40,41,42].

In addition to the immune molecules and specialized nutrients in human milk, a complex microbiota is a key component of MOM that is severely affected by the pasteurization process. In a recent study, we observed a large variability in bacterial content among mothers and between DHM and MOM samples. These differences may reflect variability in several factors, including mother’s genetic background, gestational age at delivery, stage of lactation, diet, HMO secretor status and mother’s geographic origin [40,41,42,43]. Lower counts of culturable bacteria were determined from DHM samples than MOM samples. In this study, we propose that the microbiota in DHM may be restored by the addition of small amounts of MOM. We tested increasing concentrations of MOM (1, 5, 10 and 30%) and incubation at 37 °C for 4 and 8 h. It was determined that inoculation of 30% *v*/*v* (MOM into DHM), resulting in personalized milk 30% (RM30), followed by incubation for 4 h at 37 °C, resulted in the expansion of nearly 50% of the bacterial families found in MOM [43].

In this report, we compare the global metabolite profiles of MOM and DHM, and further determine the impact of microbial expansion in RM samples on the global metabolomic profile of MOM and DHM.

## 2. Results

### 2.1. Milk Personalization Results in Global Shifts in the Metabolite Pools

Previously, we showed that personalization of DHM, with MOM (RM), allowed for the expansion of nearly 50% of the microbiota present in MOM [43]. Here, we determined the impact of the microbiota expansion on the global metabolite composition. We performed untargeted High-Performance Liquid Chromatography-High Resolution Mass Spectrometry (UHPLC-HRMS) on eight sets of DHM samples and their respective RM10, RM30 and MOM. Samples were analyzed before and after 4 h of incubation at 37 °C (T0 and T4, respectively).

**Figure 1:**
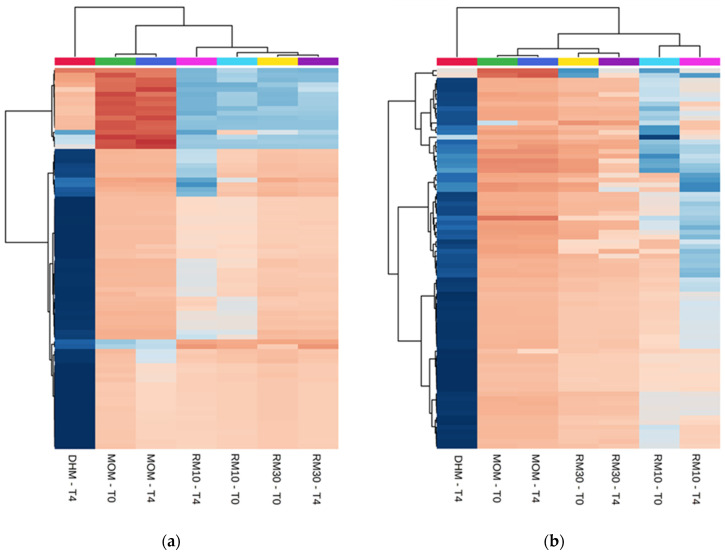

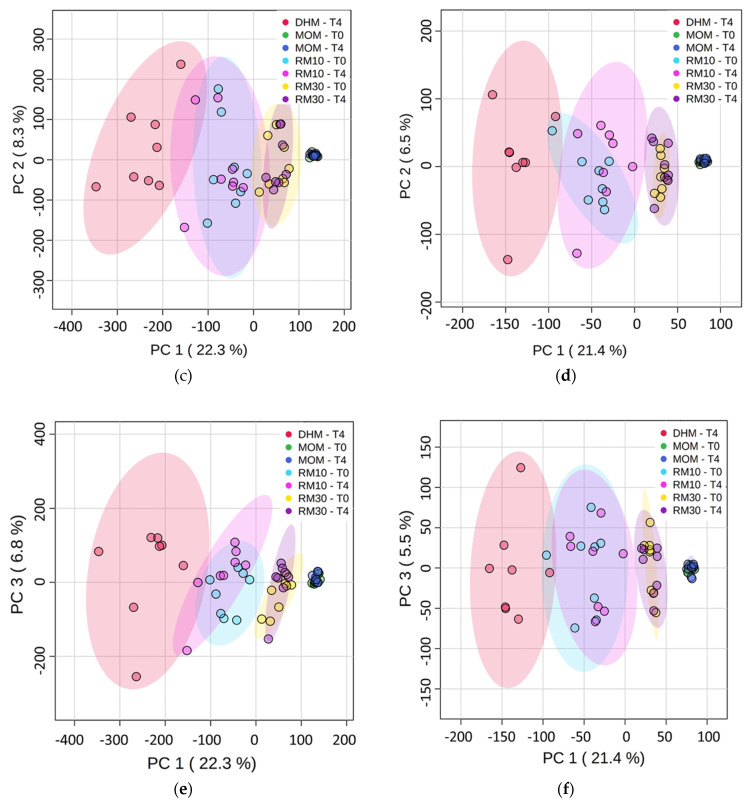
Differentially abundant metabolites. (**a**,**b**) Heatmap of the statistically significant (*p* < 0.05) differential metabolites with fold change >2.0 (Top-80 features). One-way analysis of variance (ANOVA) was performed to compare the concentration of the features in each group. (**a**) Positive ion detection mode features (**b**) Negative ion detection mode features. (**c**–**f**) Principal Component Analysis plots (**c**) PC1 vs. PC2 and (**e**) PC1 vs. PC3 for Positive ion detection mode data (4794 metabolites). (**d**) PC1 vs. PC2 and (**f**) PC1 vs. PC3 for Negative ion detection mode (1837 features). The data was auto scaled before PCA was performed. Time points are indicated for samples analyzed before and after 4 h of incubation at 37 °C (T0 and T4, respectively). Color coding: red-DHM; green and blue-MOM; light blue and pink-RM10; yellow and purple-RM30 samples. The shaded ovals are the 95% data ellipses.

The metabolomics dataset consisted of 6631 features detected between positive and negative ion mode. For this experiment, different batches of DHM were used for each set of DHM/MOM microbial expansion experiments. To minimize the variability contributed by DHM, the concentration of metabolites was normalized within each DHM/MOM/RM10/RM30 set to the corresponding DHM at T0, as well as to the dilution effect of MOM (10% in RM10 or 30% in RM30). Significant differences were found between MOM and DHM metabolite profiles, with RM samples clustering in MOM samples (Figure 1a,b). Principal Component Analysis (PCA) illustrated a clear cluster separation into MOM, RM30, RM10 and DHM samples, which accounted for 22% of the variance seen in metabolites detected on positive and negative ion mode (Figure 1c,d). The feature concentrations in DHM had a larger variability when compared to features in MOM, which clustered in a homogeneous group. The analyses of “non normalized” DHM samples (T0 and T4) showed that the variability observed is due to the heterogeneous nature of the DHM samples and not due to microbial activity during the incubation time (Supplementary Materials Figure S1a). Additionally, while PC1/PC2 allowed the separation into treatment groups (MOM dilutions into DHM), the variance in PC3 was explained by the microbial expansion observed after 4 h of incubation (Figure 1e). Using a PLS-Discriminant Analysis (PLS-DA) it was possible to emphasize group structure and discriminate groups over time in RM30 (Figure S2).

**Figure 2:**
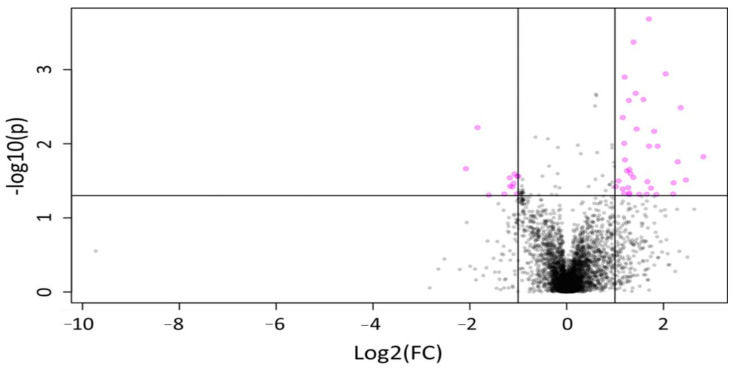
Volcano plot representing changes in the concentration of features over time in MOM samples for both ion detection modes. A total of 81 features showing a statistically significant difference (*p* < 0.05) in relative intensity after 4 h of incubation (pink circles), with 69 showing elevated expression over time.

Next, we evaluated the changes in metabolite composition after 4 h of incubation, at 37 °C in MOM, to establish a baseline of the microbial contribution to the metabolite pools. It was found that the concentration of 69 features increased significantly (*p* < 0.05) after 4 h of incubation in MOM samples (Figure 2; Table S1). As expected, no significant differences were found over time in the concentration of the features quantified in DHM. These results indicate that the fluctuations in feature concentrations observed in MOM may be due to microbiota expansion; however, most of those compounds remain unknown, as their retention time and mass-to-charge ratio (*m*/*z*) did not match any characterized compound in the different databases available online (HMDB, PubChem, CEU, or CAS Registry).

Similar to MOM, analyses of the RM10 and RM30 metabolomic profiles from samples collected after 4 h of incubation, showed a significant increase (*p* < 0.05) in the concentration of 54 and 22 features in RM10 and RM30, respectively, after the microbiota expansion (Tables S2 and S4).

While several metabolites increased in concentration as a result of microbial expansion in RM10 (Table S4), we were not able to identify them. Interestingly, RM10 and MOM samples share a unique compound (304.0792 *m*/*z*) which increased in concentration by 3.3- and 2.4-fold in RM10 and MOM, respectively. Microbial expansion in MOM and RM30 resulted in a significant (*p* < 0.05) increase in 4,8 dimethylnonanoyl carnitine (2.3- and 6.1-fold, respectively). This compound is an intermediate in the metabolism of fatty acids, phytanic and pristanic acids [44]. While this metabolic pathway has been studied in depth in mammalian cells, it has not been thoroughly studied in bacterial systems; in the literature, it is only described in *Sphingomonas paucimobilis* and marine bacterial communities [45,46,47].

During microbial expansion in MOM, six additional compounds were identified that significantly increase in concentration (*p* < 0.05). Of those, phosphatidylcholine and dihydroxyacetone phosphate (DHAP) are the only two metabolites that have been previously reported as being a result of microbial metabolic activity. DHAP is an intermediate in glycerol metabolism and was found to increase by 3.3-fold in MOM samples (*p* < 0.001). It has been reported that several bacterial species found in milk, including *Lactobacillus plantarum, L. delbrueckii, L. acidophilus, L. rhamnosus*, and *Enterococcus faecalis*, are able to catabolize glycerol into DHAP through their metabolism [48,49]. It has also been reported that gut microbiota can use choline as a precursor to produce phosphatidylcholine [50,51]. Interestingly, phosphatidylcholine increased by 5.6-fold (*p* < 0.05) in MOM after microbial expansion. Other compounds that significantly increased in MOM, but have not been previously described as products of bacterial metabolic activity, include: D-Glucaro-1,4-lactone (1,4-GL), 4.8 fold; 10,11-Dihydro-12R-hydroxy-leukotriene E4, 2.4 fold; 2-propenyl propyl disulfide, 2.0 fold.

In summary, while we were able to identify the chemical structure of several features, the majority of metabolites that were found to increase in concentration over time in the RM samples, indicates the presence of an active microbiota expansion which may contribute to the rich constitution of the milk metabolome.

### 2.2. Microbiota Expansion Does Not Affect the Concentration of the Most Abundant Metabolites in Milk

Next, we investigated whether the microbiota expansion in DHM would result in the depletion or further decrease in concentration of metabolites in milk. A total of 184 metabolites were identified in all samples by *m*/*z* and retention time (Table S3). Within the most abundant compounds, we identified the three water-soluble choline forms (free choline, phosphocholine and glycerophosphocholine) which contribute over 80% of the total choline in MOM [52,53,54,55,56]. Choline is known as an essential nutrient with several key roles in growth, brain function, and neurodevelopment. Some of the most important biological roles of this compound include neurogenesis and synapse formation, membrane biogenesis, and cell division [57,58,59]. While there was no significant increase or decrease in concentration observed for the water-soluble choline forms after 4 h of incubation in any of the samples, lower levels of choline derivatives were found in most DHM samples when compared to MOM and RM samples (Table S5).

The concentration of several amino acids (tryptophan, proline, phenylalanine, leucine, tyrosine, and isoleucine) also showed no significant changes among the different sets of samples, before or after the 4 h incubation. Nonetheless, higher variability was observed in the concentrations of glutamine, one of the most abundant amino acids in human milk.

We identified several sugars within the most abundant components; no significant differences between DHM/MOM/RM samples were observed in the concentrations of lactose, inositol, glucose, or galactose (Table S3). Citrate was identified as the most abundant intermediate of the tricarboxylic acid energy cycle, followed by malate, succinate, and isocitrate. Despite the citrate concentration being higher in MOM compared with DHM, the differences were not statistically significant (Table S3). In addition, as a result of the incubation process, the main HMO, Lacto-n-fucopentaose I (LNFP), was found at 2.2 and 3.8-fold higher concentration in RM30 and MOM samples respectively, when compared to DHM (*p* = 0.001).

In summary, these results indicate that the personalization process does not result in significant changes in the most abundant metabolites in MOM.

### 2.3. Microbiota Expansion in DHM Results in Metabolites that Modulate Similar Host Networks to MOM

**Figure 3:**
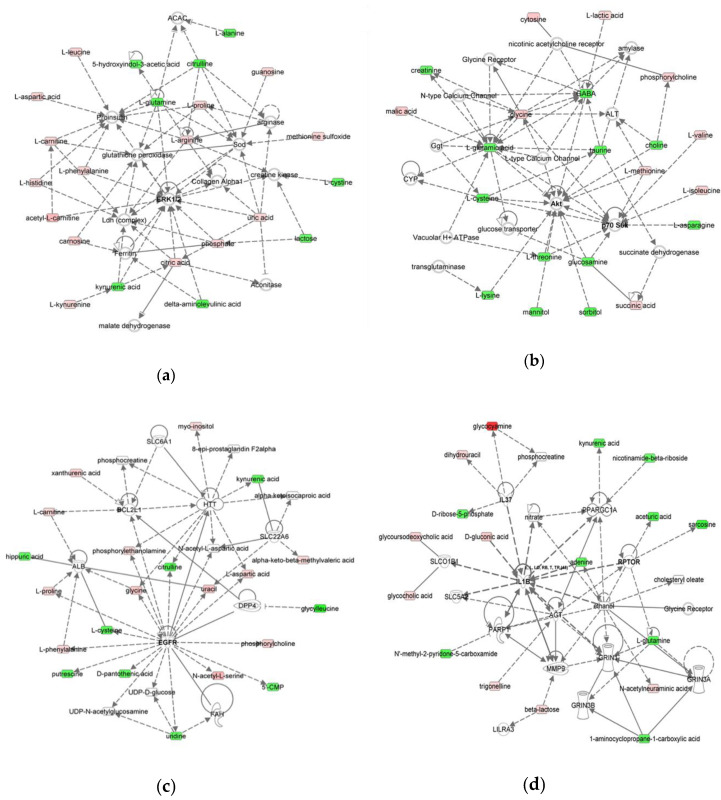
Molecular Networks predicted to be modulated by MOM and RM. The networks were obtained by analyzing the differentially expressed metabolites (Table S3) using IPA. Identified deregulated metabolites involved in the network are represented in green (decrease of concentration) and red (increase of concentration) colors. (**a**) Network 1: cellular compromise, lipid metabolism, small molecule biochemistry, (**b**) Network 2: cellular growth and proliferation, (**c**) Network 3: inflammatory response, and (**d**) Network 4: nucleic acid metabolism, amino acid metabolism, carbohydrate metabolism, molecular transport.

To evaluate whether the microbial expansion-mediated fluctuation in the metabolome might affect host pathways, Ingenuity Pathways Analysis (IPA) software was used to compare and integrate the data obtained from these samples. Four host networks were predicted to be differentially affected by the microbial expansion in MOM and RM samples: (1) cellular compromise, lipid metabolism, small molecule biochemistry; (2) cellular growth and proliferation; (3) inflammatory response; and (4) nucleic acid, carbohydrate, and amino acids metabolism, and molecular transport (Figure 3; Table S6).

The metabolite levels for each group and timepoint were compared to DHM at T0. Comparing the overall expression of the metabolites identified, similar profiles were observed among MOM and RM samples. After 4 h of microbial expansion, it was found that RM30 showed a similar profile to MOM at T0 (Table S6).

**Figure 4:**
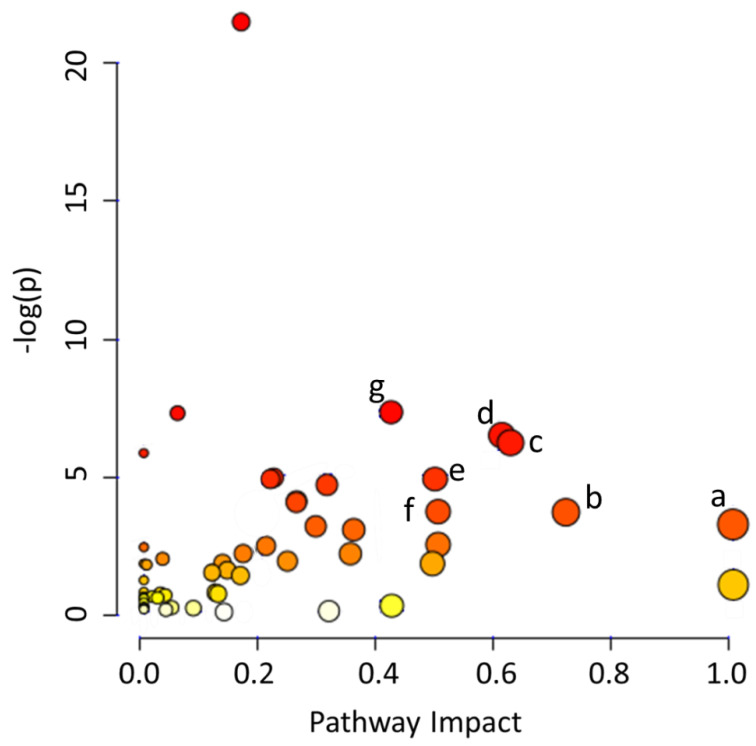
Identification of the main metabolic pathways that were determined to be impacted by microbial activity in MOM and RM samples using MetaboAnalyst 4.0. The metabolic pathways significantly impacted by fluctuations in concentration of the different features are listed as: (**a**) phenylalanine, tyrosine and tryptophan biosynthesis (*p* = 0.042), (**b**) taurine and hypotaurine metabolism (*p* = 0.028), (**c**) alanine, aspartate and glutamate metabolism (*p* = 0.002), (**d**) glycine, serine and threonine metabolism (*p* = 0.002), (**e**) tryptophan metabolism (*p* = 0.008), (**f**) ascorbate and aldarate metabolism (*p* = 0.028), and (**g**) arginine biosynthesis (*p* < 0.001).

Three of the four networks identified are directly related to cellular metabolism, supporting the expansion of the microbiota and the hypothesis that its restoration may have an impact on the composition of milk without affecting the concentration of the main metabolites. Using MetaboAnalyst 4.0, we determined that the main metabolic pathways impacted were: (a) phenylalanine, tyrosine and tryptophan biosynthesis (*p* = 0.041), (b) taurine and hypotaurine metabolism (*p* = 0.027), (c) alanine, aspartate and glutamate metabolism (*p* = 0.002), (d) glycine, serine and threonine metabolism (*p* = 0.002), (e) tryptophan metabolism (*p* = 0.008), (f) ascorbate and aldarate metabolism (*p* = 0.027), and (g) arginine biosynthesis (*p* < 0.001) (Figure 4; Table S7). In summary, we found evidence of an active metabolism due to microbiota expansion. Our data indicates that the microbial restoration in DHM may result in the modulation of similar pathways observed in MOM, in the host.

## 3. Discussion

In this study, we found that the MOM metabolomic profiles were significantly different (*p* < 0.05) than DHM and RM sample profiles, which clustered in separate groups. The DHM profile also exhibited wide variability among the samples. Variability in DHM metabolomic profiles can be explained first by DHM composition, since it is composed of a pool of mothers’ milk [60]. A second major contributor is the pasteurization process since most of the compounds may suffer different grades of inactivation/degradation during the heat treatment. We identified several compounds that increased in concentration after four hours of incubation, including 69 compounds in MOM, 54 in RM10, and 22 in RM30 samples. The absence of viable microbiota in DHM was shown by the absence of changes in the metabolites profile after 4 h of incubation. It is known that several enzymes are present in breast milk, such as amylases, lipases, proteases, lactoferrin and lysozymes that might not be active in DHM [61]. However, the great variation observed after 4 h of incubation can be better explained by the bacteria expansion of one logarithmic unit, as previously described by Cacho et al. (2017). Using a PLS-DA, we were able to emphasize group structure and discriminate sample groups over time after the inoculation of DHM with 30% MOM (RM30) [62]. PLS-DA comparing DHM and RM30 features showed a clear clustering over time in RM30 samples, suggesting that the variation of the feature concentrations may be due to the microbiota expansion as determined in our previous study [43]. No significant differences were detected in DHM samples over time. DHM is carefully collected, homogenized, and further pasteurized using a Holder method to ensure safety standards [63]. This support our hypothesis that microbiota expansion could be contribution in the variation observed in the metabolomic profiles of MOM and RM samples. The most abundant genera found in MOM samples were *Halomonas*, *Staphylococcus*, *Shewanella*, *Corynebacterium*, *Enterobacteriaceae* genus, *Acinetobacter*, unclassified *Methylobacteriaceae* genus, unclassified *Enterobacteriaceae* genus, *Bacteroides*, *Stenotrophomonas*, and *Lactobacillus*. Except for the skin commensal *Staphylococcus*, no core microbiome expansion was determined in RM samples. Similar to the microbiome outcomes, the metabolome analyses highlighted a personalized pattern of fluctuating metabolites in MOM and RM samples. Microorganisms are incredibly versatile, and different species have developed a wide variety of metabolic pathways to live and thrive within a single niche. Consequently, a varying array of metabolites may be found as end products during the microbial expansion process in different MOM samples.

One of the goals of this study was to test if the personalization process would have an impact on the most abundant nutritional components found in human milk. In this study, we were able to identify a total of 184 chemical features, which included sugars, amino acids, organic acids, lipids and lipid like-molecules, nucleotides, and vitamins (Table S3). Few of the compounds identified showed significant differences in concentration after the personalization process, when compared to DHM. The most significant differences were observed when comparing MOM to DHM. For example, lower levels of choline and choline derivatives, an important mediator of brain development, were found in DHM when compared to MOM and RM samples. As indicated previously, DHM requires safe handling procedures, where mature milk from different mothers is pooled, pasteurized, cooled, and stored in small containers. The pasteurization process can explain itself the lower concentrations of choline derivatives observed in our analysis. Moukarzel (2019) reported that the pasteurization process resulted in a significant decrease (5%) in choline concentrations [64]. Sundekilde and colleagues (2016) also reported a significant difference in phosphocholine concentration between full term human milk (the source of DHM) and preterm milk, with preterm milk with the highest concentration [65].

We did not find significant differences in the concentration of amino acids during the personalization process, except for glutamine, which was found at higher concentrations in DHM when compared to MOM. Since mature milk is the likely source of DHM, our results agree with previous reports that glutamine levels gradually increase over the first few weeks after birth, reaching its highest levels in mature milk [66,67].

Carbohydrates are the major macronutrient in human milk and contribute over 60% of the energy in MOM [68,69]. Concentrations of glucose were positively associated with relative weight and both fat and lean mass of breastfed infants [70]. Lactose accounts for approximately 85% of the total carbohydrates in MOM [71] and is often considered to be the total carbohydrate content in human milk. In this study, we did not find significant differences between the samples; however, the concentrations found are in concordance with previous reports. We also observed decreased levels of citric acid in DHM when compared to MOM, in agreement with the report that citric acid concentration decreases significantly over time, postpartum [72].

In this study, the concentration of LNFP was 3.5 times higher in RM30 and MOM samples than in DHM (*p* < 0.001). LNFP is one of the most abundant oligosaccharides in milk [73]. Since HMOs cannot be metabolized by human cells, the most important function assigned to HMOs is the ability to modulate the microbiota composition by selective enrichment of species that can metabolize these complex sugar moieties, thereby influencing several physiological processes [74], and protecting infants from pathogenic infections [75,76]. The most prominent example of this function is the enrichment of *Bifidobacterium* species [77]. Other beneficial effects of HMOs include the ability to reduce *Streptococcus pneumoniae* adherence to cells during upper respiratory infections [78] as well as *Escherichia coli* gastrointestinal adherence [79,80,81]. The higher concentration of LNFP in MOM and RM30 samples suggested that LNFP may be sensitive to pasteurization, in contrast to previous reports [82,83]. Our findings indicate that the expansion of the microbiota does not have a detrimental effect on the levels of this important group of sugars. A limitation of our study; however, is that approximately 200 types of HMOs have been identified [84,85] and further analysis, using specialized extraction techniques, is needed to fully evaluate the impact of the expanding microbiome. Differences in the composition of infant’s microbiome was observed depending on the “secretor” status of the mothers [42]. However, a limitation of our study is that the “secretor” status of the mothers was not investigated here, and should be considered in future studies in order to determine if there is a link between the “secretor” status and MOM microbiome that could have an impact in the metabolome composition of MOM.

We were able to identify several compounds that may be used to monitor microbial expansion. Phosphatidylcholine and DHAP significantly increased in concentration in MOM, while the concentration of 4,8 dimethylnonanoyl carnitine increased in MOM and RM30. The beneficial roles of these compounds and/or their derivatives metabolism has been reported in mammal cells [45,46,47]. For example, 4,8 dimethylnonanoyl carnitine is an intermediate in the metabolism of phytanic and pristanic acids. Phytanic acid is one of the most common branched fatty acids in the human diet, and it can be used as an indicator of ruminant and fish-derived fat consumption [86]. The consumption of this fatty acid has several beneficial effects on the body, such as its involvement in glucose metabolism [87,88], prevention of metabolomic syndrome [89], and immunomodulatory effects [90]. Similarly, Simone and colleagues (2001) found that the oral supplementation of 1,4-GL resulted in antiproliferative effects and helped to control the progression of breast, prostate, and colon cancers [91]. In addition, it was found that the administration of 1,4-GL resulted in modifications in the gut microbiota in a rodent model, notably decreasing *E. coli* and increasing *Bifidobacterium* and *Lactobacillus* species [92].

Phosphatidylcholine can be found in MOM at a concentration of 2.8 mg/100 g [93]. This phospholipid is one of the most abundant in human milk and contributes around 40% of the total phospholipid content (1–2% of total lipids in milk) [94,95]. The increase in concentration of phosphatidylcholine during microbial expansion in MOM is highly significant since it represents 10% of the total choline intake of infants. This essential nutrient has several important biological roles that are crucial for neonatal development, including neurogenesis and synapse formation, membrane biogenesis, and cell division [58,59]. These findings highlight the importance of microbial metabolic activity as a significant contributor to the increase of known bioactive compounds in human milk.

Finally, we identified four host networks that are impacted by the fluctuation in metabolites identified in MOM, DHM and RM. Three of the four networks identified are directly related to cellular metabolism and cell survival. Notably, many proteins involved in the mTOR signaling cascade (ERK1/2, Akt, p70-S6K, EGFR, IL37, Il1B, and RPTOR) are impacted by the shifts in metabolite concentrations. The mTOR pathway is involved in the regulation of cell growth and proliferation, as well as survival. The regulation of this pathway is complex and is highly dependent on the cell’s environment, with profound impacts in cell senescence and in immune regulation (for a review see [96]). The amino acids leucine and arginine are two activators of the mTOR pathway [97,98]. Interestingly, we did not observe significant changes in amino acid concentrations when analyzed individually, but as part of a complex metabolome, the fluctuations in metabolites significantly impacted these pathways. MOM has been shown to provide bioactive components that function synergistically to ensure the infant’s protection by acting as immunomodulators, and pasteurization significantly affects their availability [99,100,101,102,103,104]. However, further experiments need to be performed in order to understand the impact of RM on the mTOR pathway and the consequences on immune regulation in response to an active microbiome.

In summary, the metabolomes of DHM, MOM and RM samples analyzed in this study showed significant changes in their composition following expansion of the microbiota. We found that while no significant changes were observed in concentration of the most abundant sugars and amino acids, we were able to successfully detect 69 compounds in MOM and 22 in RM30 that changed significantly as a result of the microbial activity. We also identified at least three compounds that may be used as biomarkers of microbial expansion. A limitation of this study is the inability to identify the chemical nature of most of the compounds that changed during microbial expansion; however, we expect to be able to use these “unknown” metabolites to track microbial expansion in RM samples in the near future, as the metabolomics field continues to expand.

## 4. Materials and Methods

### 4.1. Experimental Design

The samples analyzed in this study are a subset of those collected during the study reported [43]. While 12 samples were used in the original study, four of those sets were no longer available for this study and hence excluded from the analyses. Demographics are described in Table 1. Briefly, for the microbial restoration, increasing amounts of MOM (10% and 30% *v*/*v*) were added to pasteurized DHM. Pure DHM and MOM were included as controls. The milk mixtures were incubated at 37 °C. Samples for metabolome study and further studies were taken at time 0 and 4 h. For exclusion criteria and further details of the microbiota restoration strategy, see Cacho et al., 2017.

### 4.2. Metabolites Extraction

The metabolites were extracted from each sample as previously described [105] using 10 µL of 2× diluted Metabolomics IS Mix and 200 µL of 8:1:1 Acetonitrile/Methanol/Acetone, and incubated for 30 min at 4 °C. The samples were centrifuged at 20,000× *g* at 4 °C for 10 min, and 190 µL of the supernatant was dried under nitrogen gas at 30 °C. All samples were stored at −80 °C. Samples were reconstituted to 25 µL in 0.1% formic acid, and analyzed on a Q Exactive™ Hybrid Quadrupole-Orbitrap High Resolution Mass Spectrometer (Thermo Fisher Scientific, San Jose, CA, USA). The metabolomics injection standards mix is constituted by Leucine-13C6 (4 µg/mL), Creatine-D3 H2O (methyl-D3) (4 µg/mL), L-Leucine-D10 (4 µg/mL), L-Tryptophan-2,3,3-D3 (40 µg/mL), L-Tyrosine Ring-13C6 (4 µg/mL), L-Phenylalanine Ring-13C6 (4 µg/mL), N-BOC-L-tert-Leucine (4 µg/mL), N-BOC-L-Aspartic Acid (4 µg/mL), Propionic Acid 13C3 (8 µg/uL), Succinic Acid-2,2,3,3-d4 (8 µg/mL), Salicylic Acid D6 (4 ug/mL), Caffeine-d3 (1-methyl-d3) (4 µg/mL) and was reconstituted in 0.1% formic acid. A Red Cross Plasma positive control and an extraction blank negative control were extracted as Quality Controls (QC). A Neat QC was made with 1:1:3 Metabolomics IS Mix/Amino Acids Mix/0.1% formic acid.

### 4.3. Analytical Instrumentation and Methodology

Global untargeted metabolomic analyses by UHPLC-HRMS were performed on a Thermo Q Exactive Orbitrap Mass Spectrometer, with heated electrospray ionization source coupled to a Dionex Ultimate 3000 UHPLC system (Thermo Scientific, Waltham, MA, USA). Reverse phase chromatography with gradient elution was employed using an ACE Excel 2 C18-PFP column (100 mm × 2.1 mm, 2.0 μm) (Advanced Chromatography Technologies, Ltd., Aberdeen, Scotland). Gradient elution was performed with 0.1% formic acid as solvent A and acetonitrile as solvent B, at a flow rate of 0.35 mL/min, as follows: 0–3 min: 100% A, 3–13 min: 100% → 20% A, 13–16.5 min: 20% A, 16.5–20 min: 100% A at 0.6 mL/min (column flush & equilibration). Injection volume was 5 μL. Data were acquired in both positive and negative ion mode (separate injections) by full scan analysis from *m*/*z* 70–1000 at 35,000 mass resolution.

### 4.4. Data Normalization and Statistical Analysis

MetaboAnalyst 4.0 was used for statistical analysis and figure generation [106]. Prior to data analyses, each MOM/RM10/RM30/DHM set was normalized to the concentration found in the corresponding DHM at T0 and adjusted to the dilution rate of MOM in order to minimize the variability contributed by DHM samples (10% or 30%). One-way analysis of variance (ANOVA) and t tests were used to examine the differences in metabolite concentrations. Principal component analysis (PCA) and partial least squares–discriminant analysis (PLS-DA) were performed to distinguish differentially enriched metabolic profiles in each group. In this report, we define significance with a *p*-value threshold of ≤0.05. The potential impact of the identified metabolites on the host was evaluated using MetaboAnalyst 4.0 and the software Ingenuity Pathways Analysis (IPA) (Ingenuity Systems^®^, Redwood City, CA, USA). For this analysis, the concentration found in DHM at T0 was used as the baseline. Networks with scores greater than 30, with at least 15 compounds identified in each network, were selected.

### 4.5. Metabolite Identification

For identification of the features, the mass-to-charge ratios and ionization mode were used to search on-line databases such as the Human Metabolome Database [107], CEU [108], PubChem [109] and CAS Registry [110].

## Figures and Tables

**Table 1:** Demographics.

**Infant Demographics (n = 8)**	
Gestational age at birth (weeks)	26 ± 2.67
Birth weight (grams)	831.38 ± 321.08
Post-menstrual age at sample collection (weeks)	34 ± 26.30
**Gender**	
Male	62% (n = 5)
Female	38% (n = 3)
**Delivery Mode**	
C-section	38% (n = 3)
Vaginal	62% (n = 5)
Maternal antibiotics	88% (n = 7)

## Supplementary Materials

The following are available online, Figure S1: Principal Component Analysis Plots for Negative ionization mode, Figure S2: PLS-Discriminant Analysis Plots, Figure S3: Schematic representation of the personalization process, Table S3: Compounds Identified, Table S4: Compounds Significantly Increased in RM30 Over Time (T4/T0), Table S5: Concentration of glutamine and choline derivative forms, Table S6: Molecules Regulation and Top Functions Implicated, Table S7: Pathway Impact.
