# Supplementary material for: Metabolomic Profile of Personalized Donor Human Milk
Source: Molecules. 2020 Dec 8;25(24):5783. doi: 10.3390/molecules25245783 (PMC7763631; doi:10.3390/molecules25245783)
Supplement: Supplementary file 1 [file molecules-25-05783-s001.pdf]

Supplementary Materials:

Figures

Figure S1

(a) (b)

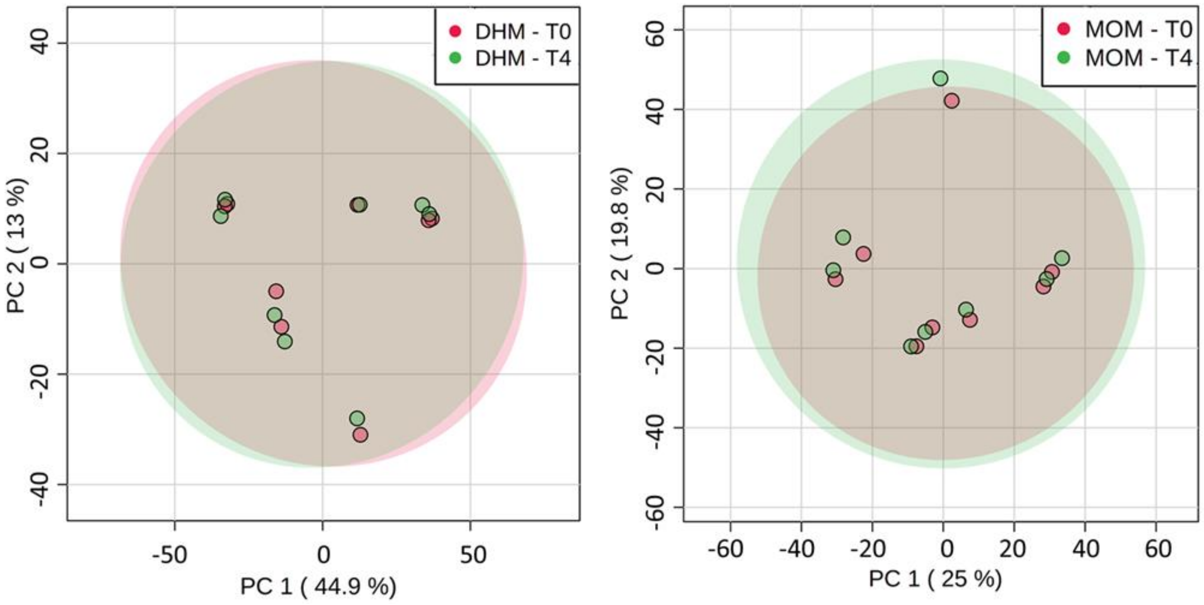

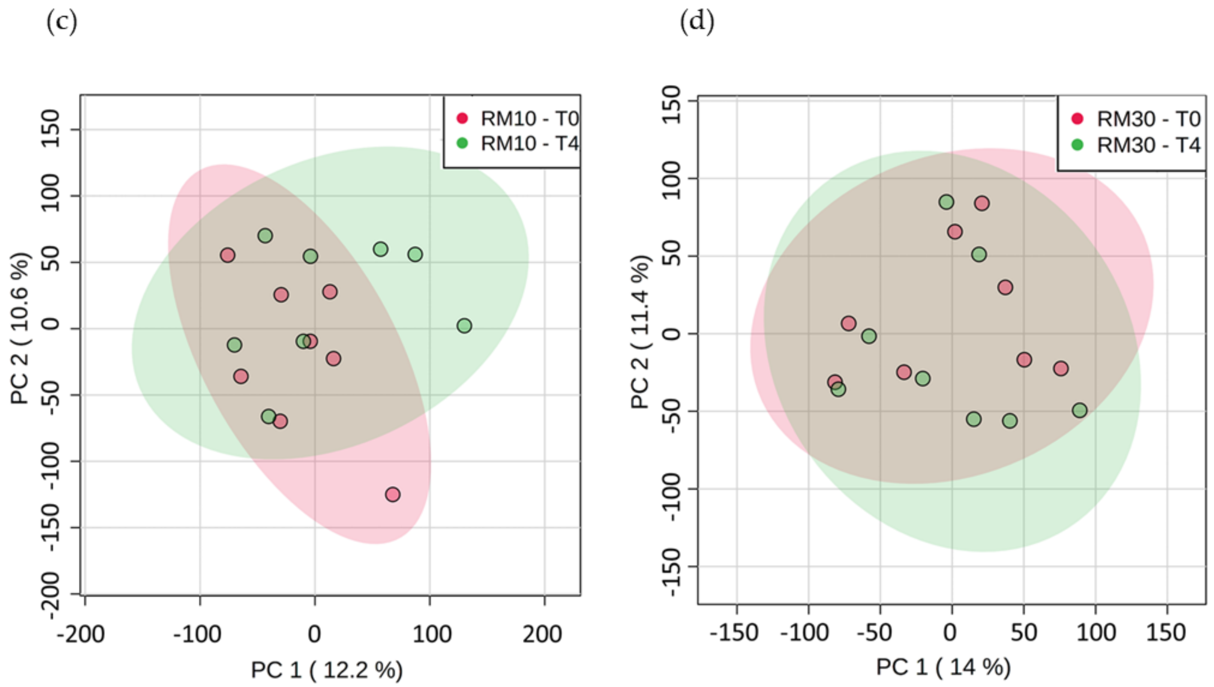

Figure S1. Principal Component Analysis Plots for Negative ionization mode (184 metabolites), Positive ionization mode showed the same patterns. (a) PC1 vs PC2 DHM features. (b) PC1 vs PC2 MOM features. (c) PC1 vs PC2 RM10 features. (d) PC1 vs PC2 RM30 features. The data were auto scaled before PCA was performed. T0: time zero; T4: time 4 hours. The shaded ovals are the 95% data ellipses.

Figure S2

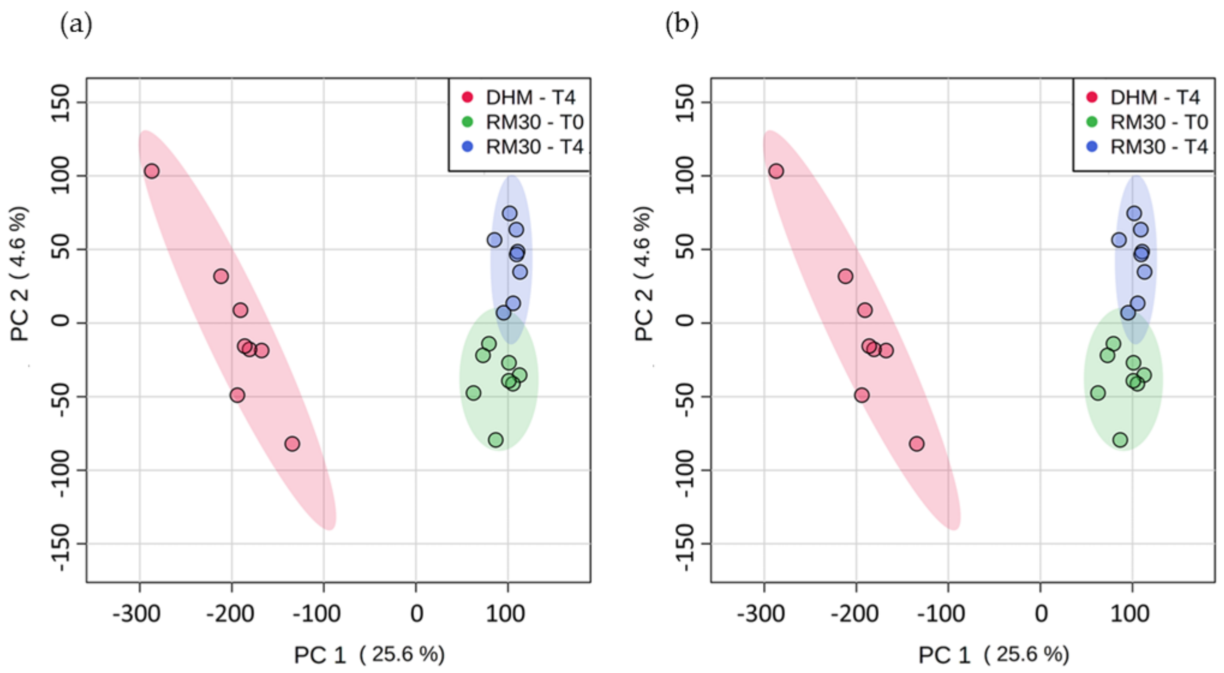

Figure S2. PLS-Discriminant Analysis Plots (a) for Positive ion mode (cross-validation measure q2 = 0.5058). (b) for negative ion mode (cross-validation measure q2 = 0.4453). T0: time zero; T4: time 4 hours.

Figure S3

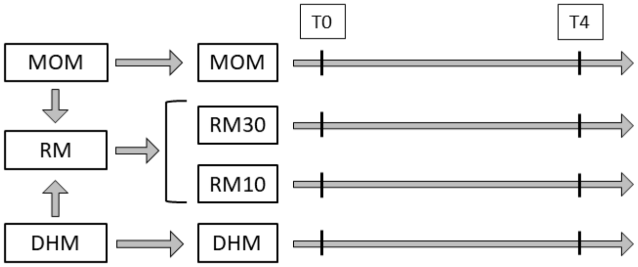

Figure S3. Schematic representation of the personalization process.

Figure S4

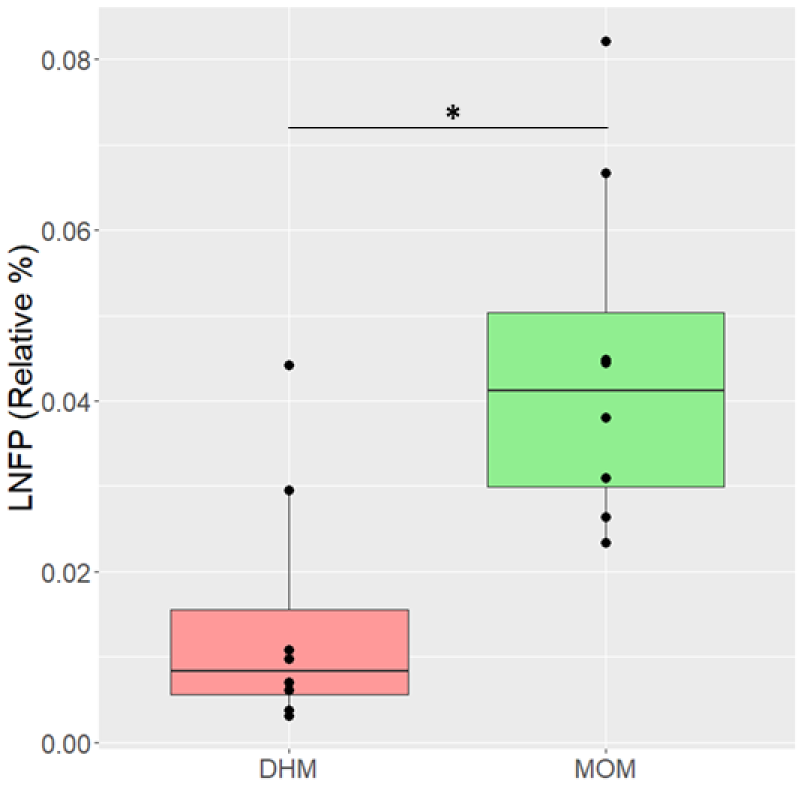

Figure S4. Relative concentration of LNFP at T0 in DHM and MOM (p<0.05).

Tables

Table S1. Compounds significantly increased in MOM over time (T4/T0). T-test was used to compare means.

	m/z	Retention time	Name	ID	Fold Change	p-value
P o si ti	105.5074	1.49	Unknown	-	2.48	0.0022

	126.021	1.49	Unknown	-	2.76	0.0017
	131.0338	1.5	dihydroxyacetone phosphate	HMDB0011675	3.33	0.0002
	132.0369	1.53	Unknown	-	2.64	0.0006
	134.0271	1.2	Unknown	-	6.89	0.0126
	136.0615	7.42	Unknown	-	2.39	0.0177
	136.0617	1.51	Unknown	-	2.65	0.0288
	174.1489	7.05	4,8 Dimethylnonanoyl carnitine	HMDB0006202	2.30	0.0016
	188.039	2.84	Unknown	-	2.13	0.0278
	190.1436	7.96	Unknown	-	2.32	0.0056
	193.0343	0.84	D-Glucaro-1,4-lactone	HMDB0041862	4.81	0.0462
	196.0464	2.29	Unknown	-	2.36	0.0175
	214.986	1.5	Unknown	-	3.09	0.0019
	225.9792	1.5	Unknown	-	3.27	0.0120
	233.1128	2.48	Unknown	-	2.83	0.0054
	238.093	6.08	8-[(Aminomethyl)sulfanyl]-6-sulfanyloctanoic acid	HMDB0013639	2.43	0.0350
	289.0548	2.8	Unknown	-	2.53	0.0485
	298.0964	7.42	Unknown	-	3.22	0.0272
	300.0909	7.42	Unknown	-	3.19	0.0409
	343.1228	2.78	Unknown	-	2.30	0.0064
	346.1173	6.85	Unknown	-	5.32	0.0024
	346.1174	6.95	Unknown	-	4.78	0.0287
	348.2736	10.61	Unknown	-	2.03	0.0210
	352.2168	11.2	Unknown	-	2.52	0.0200
	353.2293	10.61	Unknown	-	2.09	0.0341
	353.6818	8.53	Unknown	-	3.57	0.0064

	362.2892	10.61	Unknown	-	4.27	0.0011
	369.1932	10.61	Unknown	-	2.99	0.0439
	386.1284	1.91	Unknown	-	5.14	0.0143
	391.0838	1.6	Unknown	-	3.58	0.0426
	431.7421	8.14	Unknown	-	3.03	0.0472
	458.2571	8.54	10,11-Dihydro-12R-hydroxy-leukotriene E4	HMDB0012501	2.44	0.0358
	498.9371	8.25	Unknown	-	3.64	0.0087
	509.1599	1.64	Unknown	-	2.51	0.0195
	572.329	8.56	Unknown	-	2.31	0.0416
	667.3946	9.1	Unknown	-	3.33	0.0315
	679.1259	9.37	Unknown	-	2.36	0.0430
	686.8823	8.53	Unknown	-	2.01	0.0447
	702.7815	9.68	Unknown	-	3.69	0.0412
	942.6967	10.33	phosphatidylcholine	HMDB0008683	5.64	0.0284
**Negative ionization mode**	109.041	1.66	Unknown	-	2.37	0.0178
	124.9917	0.82	Unknown	-	2.06	0.0117
	129.0197	0.76	2-Propenyl propyl disulfide	HMDB0033912	2.00	0.0012
	129.0197	0.83	2-Propenyl propyl disulfide	HMDB0033912	2.04	0.0011
	130.0229	0.75	Unknown	-	2.40	0.0019
	130.0229	0.85	Unknown	-	2.23	0.0016
	134.0479	1.53	Unknown	-	3.05	0.0080
	191.0202	0.78	Unknown	-	2.05	0.0027
	191.0202	1.03	Unknown	-	2.83	0.0050
	192.0235	0.8	Unknown	-	2.24	0.0049
	192.0235	0.87	Unknown	-	3.44	0.0019
	193.0247	0.91	Unknown	-	3.50	0.0011

	228.0959	1.76	Unknown	-	2.90	0.0047
	228.0995	1.77	Unknown	-	2.91	0.0047
	234.0261	0.77	Unknown	-	2.70	0.0452
	242.0787	1.66	Unknown	-	2.05	0.0309
	248.0404	1.53	Unknown	-	2.66	0.0202
	273.1352	8.79	Unknown	-	2.26	0.0485
	275.0027	0.79	Unknown	-	3.70	0.0294
	278.0555	1.66	Unknown	-	2.05	0.0339
	280.0523	1.66	Unknown	-	2.04	0.0417
	288.0844	1.66	Unknown	-	2.03	0.0391
	293.0141	0.8	Unknown	-	3.09	0.0136
	304.0792	1.66	Unknown	-	2.44	0.0177
	358.0854	5.91	Unknown	-	4.00	0.0152
	378.0795	1.66	Unknown	-	2.25	0.0452
	384.1444	8.26	Unknown	-	2.33	0.0222
	408.1136	0.84	Unknown	-	4.96	0.0275
	503.1274	0.82	Unknown	-	4.68	0.0364

The compounds are grouped by ionization mode. Feature number corresponds to a unique identification obtained from MZmine. The ID codes correspond to Human Metabolome Database.

Table S2. Compounds significantly increased in RM30 over time (T4/T0). T-test was used to compare means.

	m/z	Retention time	Name	ID	Fold Change	p-value
Positive ionization mode	134.0271	1.2	Unknown	-	47.44	0.0061
	174.1489	7.05	4,8 Dimethylnonanoyl carnitine	HMDB0006202	6.31	0.0409
	178.0352	9.73	Unknown	-	2.25	0.0020
	198.1234	0.77	Unknown	-	2.37	0.0355
	217.0969	8.2	Unknown	-	2.12	0.0405
	226.0277	2.98	Unknown	-	2.28	0.0089
	281.0762	10.67	Unknown	-	2.02	0.0377
	305.7047	7.38	Unknown	-	2.08	0.0453
	324.0585	1.75	Unknown	-	4.16	0.0137
	343.1969	7.35	Unknown	-	2.43	0.0326
	343.7164	8.1	Unknown	-	10.46	0.0446
	477.7734	8.59	Unknown	-	100.00	0.0391
	557.9934	9.6	Unknown	-	19.20	0.0385
	572.2822	9.38	Unknown	-	3.82	0.0039
	573.3144	8.79	Unknown	-	2.06	0.0493
	638.8359	9.04	Unknown	-	2.53	0.0247
	734.094	8.94	Unknown	-	2.12	0.0101
	791.7298	10.25	Unknown	-	3.94	0.0242
	846.2146	9.46	Unknown	-	2.10	0.0246
	868.9902	9.68	Unknown	-	5.25	0.0374
Negative ionization mode	189.0043	2.54	Unknown	-	3.51	0.0423
	387.1161	9.03	Unknown	-	6.12	0.0482

The compounds are grouped by ionization mode. Feature number corresponds to a unique identification obtained from MZmine. The ID codes correspond to Human Metabolome Database.

Table S3. Compounds Identified.

Relative concentration of the compounds identified are listed as percentage grouped by type of molecules. Features were identified using three different databases. The ID codes corresponds to Human Metabolome Database, CAS Register Number, and PubChem data bases.

Table S4. Compounds significantly increased in RM10 over time (T4/T0). T-test was used to compare means.

	m/z	Retention time	Name	ID	Fold Change	p-value
**Positive ionization mode**	88.9526	0.58	Unknown	-	2.04	0.0030
	101.5277	0.59	Unknown	-	2.36	0.0074
	112.0505	1.04	Unknown	-	3.00	0.0400
	124.5	0.59	Unknown	-	6.61	0.0039
	130.1226	7.69	Unknown	-	11.66	0.0144
	131.0225	1.92	Unknown	-	2.61	0.0172
	136.0518	7.63	Unknown	-	2.64	0.0002
	136.5073	2.55	Unknown	-	2.07	0.0151
	148.0603	0.75	Unknown	-	2.36	0.0054
	195.1377	10.61	Unknown	-	2.32	0.0404
	203.0021	1.92	Unknown	-	6.57	0.0146
	220.5569	1.12	Unknown	-	2.22	0.0388
	241.9742	1.29	Unknown	-	2.16	0.0299
	264.0499	2.85	Unknown	-	3.23	0.0023
	324.0585	1.52	Unknown	-	43.61	0.0272
	440.2498	7.86	Unknown	-	3.10	0.0456
	458.2604	7.58	Unknown	-	2.09	0.0189
	522.2818	12.69	Unknown	-	7.57	0.0189
	543.3192	8.7	Unknown	-	2.03	0.0323
	557.9929	9.29	Unknown	-	6.75	0.0461
	602.3327	9.52	Unknown	-	2.96	0.0349
	623.6583	9.07	Unknown	-	5.72	0.0498
	628.9899	8.72	Unknown	-	4.86	0.0288

Classification	Database ID	Name	MOM T0	MOM T4	RM10 T0	RM10 T4	RM30 T0	RM30 T4	DMT0	DMT4	Row m/z	Row retention time
Amino acids	**HMDB0000062**	Creatine	7.2904	7.6652	8.3088	8.3148	8.3268	8.3279	8.4786	8.2339	132.0766	0.9
	**HMDB0000062**	Creatine	0.0175	0.0161	0.0243	0.0235	0.0200	0.0209	0.0295	0.0270	130.0621	0.92
	**HMDB0000562**	Creatinine	6.3657	6.2430	5.9819	5.8507	6.0841	5.9066	5.9702	5.8555	114.0662	0.93
	**HMDB0000562**	Creatinine	0.0020	0.0022	0.0028	0.0028	0.0025	0.0025	0.0031	0.0028	112.0523	0.94
	**HMDB0000687**	L-leucine	3.0426	3.7519	4.5146	4.8339	4.2546	4.8833	4.4626	4.6335	132.1018	2.53
	**HMDB0000687**	Leucine	0.0046	0.0060	0.0070	0.0077	0.0069	0.0075	0.0069	0.0072	130.0874	2.51
	**HMDB0000641**	L-glutamine	2.9438	2.5759	5.5961	5.5849	5.2325	5.0875	5.7968	5.8245	147.0762	0.72
	**HMDB0000641**	L-glutamine	0.1232	0.1017	0.3111	0.3074	0.2744	0.2855	0.3460	0.3384	145.0622	0.71
	**HMDB0000162**	L-proline	2.8853	3.1510	2.7611	2.9398	2.8037	3.0552	2.7927	2.8539	116.0705	0.85
	**HMDB0002393**	N-methyl-D-aspartic acid	1.6338	1.6088	2.2480	2.4735	2.2071	2.2796	2.4066	2.4549	148.0603	0.75
	**HMDB0000172**	L-isoleucine	1.2837	1.7027	1.5966	1.7073	1.5623	1.8390	1.5333	1.5739	132.1018	2.23
	**HMDB0000172**	L-isoleucine	0.0038	0.0046	0.0042	0.0053	0.0042	0.0047	0.0047	0.0054	130.0874	2.33
	**HMDB0000159**	Phenylalanine-HCOOH	0.6220	0.6839	0.8358	0.8518	0.8082	0.8688	0.8291	0.8338	120.0807	6.17
	**HMDB0000159**	Phenylalanine	2.4915	2.7272	3.3895	3.4334	3.2537	3.4749	3.3098	3.3326	166.0861	6.17
	**HMDB0000517**	L-arginine	0.4305	0.6177	0.7049	0.7961	0.6443	0.7507	0.7121	0.7694	175.1187	0.69
	**HMDB0000517**	L-arginine	0.0030	0.0052	0.0070	0.0088	0.0059	0.0080	0.0074	0.0082	173.1049	0.7
	**HMDB0000148**	L-glutamic acid	0.3891	0.3851	0.5013	0.5645	0.5054	0.5304	0.5320	0.5655	146.0463	0.76
		Threonine/homoserine	0.3341	0.3488	0.3935	0.3811	0.3874	0.3878	0.3921	0.3790	120.0654	0.72
		Alanine/sarcosine	0.2986	0.3123	0.3674	0.3655	0.3469	0.3527	0.3838	0.3662	90.0549	0.71
	**HMDB0000696**	L-methionine	0.2646	0.3449	0.2905	0.3454	0.2582	0.2695	0.2599	0.2915	150.0582	1.42
	**HMDB0000187**	L-serine	0.1529	0.1545	0.2749	0.2680	0.2345	0.2506	0.2944	0.2894	106.0498	0.69
	**HMDB0000187**	L-serine	0.0252	0.0251	0.0500	0.0527	0.0447	0.0478	0.0553	0.0573	104.0355	0.69
		L-methionine	0.0157	0.0218	0.0181	0.0221	0.0169	0.0191	0.0170	0.0191	148.0441	1.43
	**HMDB0002005**	Methionine sulfoxide	0.1333	0.1350	0.1820	0.1708	0.1772	0.2026	0.1935	0.1798	166.0528	0.78
	**HMDB0000123**	Glycine	0.0745	0.0749	0.1217	0.1180	0.1066	0.2178	0.1562	0.1258	76.0393	0.71
	**HMDB0000177**	L-histidine	0.0642	0.0675	0.1077	0.1156	0.0979	0.1105	0.1211	0.1264	154.0626	0.69
	**HMDB0000177**	L-histidine	0.6053	0.5854	0.7248	0.7387	0.6901	0.7756	0.7675	0.7725	156.0766	0.69
	**HMDB0000929**	Tryptophan	0.0549	0.0652	0.0775	0.0886	0.0744	0.0853	0.0818	0.0834	203.0832	7.71
	**HMDB0000929**	Tryptophan	0.5476	0.6510	0.7460	0.8117	0.7165	0.8135	0.7593	0.7633	205.0972	7.71
		Tryptophan-NH3	0.2497	0.2962	0.3370	0.3664	0.3227	0.3688	0.3499	0.3461	188.0705	7.71
	**HMDB0000191**	Aspartate	0.0936	0.0953	0.0880	0.0970	0.0885	0.1008	0.0894	0.1054	132.0306	0.71
	**HMDB0000168**	L-asparagine	0.0495	0.0509	0.0730	0.0739	0.0675	0.0731	0.0785	0.0765	133.0607	0.7
		Asparagine	0.0156	0.0159	0.0316	0.0322	0.0289	0.0310	0.0357	0.0356	131.0465	0.7
	**HMDB0002931**	N-acetyl-DL-serine	0.0348	0.0148	0.0227	0.0191	0.0281	0.0314	0.0170	0.0228	146.0462	1.04

	**HMDB0000996**	3-sulfino-l-alanine	0.0321	0.0289	0.0226	0.0214	0.0291	0.0248	0.0173	0.0178	152.0027	0.82
		L-tyrosine	0.0591	0.0742	0.0637	0.0689	0.0659	0.0702	0.0649	0.0666	180.067	3.42
	**HMDB0000182**	L-lysine	0.0228	0.0470	0.0564	0.0694	0.0478	0.0626	0.0630	0.0705	147.1127	0.62
	**HMDB0000251**	Taurine	0.0161	0.0225	0.0251	0.0232	0.0224	0.0274	0.0260	0.0153	124.0075	0.81
	**HMDB0000251**	Taurine	1.5021	1.4418	1.7827	1.6327	1.6487	1.6427	1.8542	1.6811	126.0219	0.7
	**HMDB0000251**	Taurine	1.1668	1.1001	1.6321	1.4982	1.4590	1.5288	1.6232	1.6104	124.0076	0.68
	**HMDB0000766**	N-acetyl-L-alanine	0.0185	0.0190	0.0167	0.0171	0.0172	0.0174	0.0169	0.0173	130.0511	2.26
	**HMDB0000742**	Homocysteine	0.0114	0.0111	0.0240	0.0247	0.0189	0.0192	0.0245	0.0247	136.0426	0.9
		Sarcosine/beta-alanine	0.0102	0.0096	0.0145	0.0147	0.0123	0.0129	0.0170	0.0165	88.0402	0.9
	**HMDB0000128**	Guanidinoacetate	0.0082	0.0182	0.0129	0.0032	0.0032	0.0176	0.0035	0.0133	118.061	0.78
	**HMDB0003374**	Ornithine	0.0054	0.0055	0.0106	0.0110	0.0079	0.0088	0.0106	0.0229	133.0971	0.61
	**HMDB0000883**	L-valine	0.0050	0.0055	0.0073	0.0081	0.0072	0.0081	0.0080	0.0083	116.0716	1.13
	**HMDB0001138**	N-acetyl-DL-glutamic acid	0.0064	0.0068	0.0060	0.0066	0.0068	0.0068	0.0064	0.0065	188.0578	2.24
	**HMDB0004620**	N-acetyl-arginine	0.0018	0.0021	0.0013	0.0013	0.0015	0.0014	0.0012	0.0012	217.1293	1.64
	**HMDB0000446**	N-alpha-acetyl-L-lysine	0.0016	0.0026	0.0012	0.0012	0.0012	0.0013	0.0010	0.0012	189.1231	1.06
	**HMDB0000472**	5-hydroxy-L-tryptophan	0.0011	0.0013	0.0011	0.0011	0.0016	0.0013	0.0009	0.0013	221.0917	6.85
**Organic acids and derivatives**	**HMDB0000094**	Citrate	2.2265	2.4329	1.5001	1.8426	1.7249	1.7771	1.5622	1.6355	191.0202	1.92
	**HMDB0000094**	Citrate	0.0899	0.0983	0.0516	0.0668	0.0609	0.0602	0.0549	0.0540	193.0341	1.91
		Citrate+Na	0.2718	0.3048	0.1426	0.1888	0.1735	0.1756	0.1516	0.1516	215.0158	1.91
		Citrate+NH3	0.2611	0.2843	0.1532	0.1948	0.1823	0.1794	0.1613	0.1596	210.0605	1.91
		Citrate source fragment (+H and + NH3)	0.2474	0.2745	0.1431	0.1795	0.1734	0.1742	0.1518	0.1517	129.0181	1.91
		Citrate source fragment (+NH3)	0.1193	0.1341	0.0693	0.0892	0.0831	0.0828	0.0722	0.0725	175.0235	1.91
		Citrate source fragment (+NH3)	0.0518	0.0580	0.0299	0.0384	0.0360	0.0360	0.0327	0.0320	157.0131	1.91
	**HMDB0000190**	L-(+)-lactic acid	0.9447	0.9277	0.8558	0.7938	0.8848	0.8337	0.8530	0.8105	91.0389	0.73
	**24277914**	2-hydroxyphenylalanine	0.7875	0.9633	0.7898	0.8353	0.7997	0.8825	0.7743	0.7813	182.081	3.4
	**HMDB0000744**	Malate	0.6555	0.6569	0.2817	0.3130	0.3543	0.3601	0.2672	0.2669	133.0146	1.07
	**HMDB0031518**	(R)-malate	0.0025	0.0022	0.0006	0.0008	0.0010	0.0009	0.0007	0.0004	157.0109	1.17
	**HMDB0031518**	(R)-malate	0.0171	0.0175	0.0069	0.0069	0.0085	0.0086	0.0067	0.0063	157.0111	1.07
	**HMDB0000254**	Succinate	0.4475	0.5258	0.6246	0.7085	0.5687	0.7095	0.6215	0.6780	117.0194	2.31
	**HMDB0000254**	Succinate	0.0114	0.0129	0.0155	0.0170	0.0138	0.0181	0.0154	0.0160	141.0161	2.3
	**HMDB0004827**	Stachydrine	0.4172	0.4352	0.3921	0.3652	0.4011	0.3953	0.3562	0.3792	144.1018	1.06
	**HMDB0000479**	N(PAI)-methyl-L-histidine	0.2982	0.2973	0.5208	0.5109	0.4818	0.5057	0.5599	0.5726	170.0922	0.69

	**HMDB0000267**	5-oxo-l-proline	0.2965	0.3484	1.0074	1.0076	0.8406	0.8692	1.0422	1.0948	130.0498	1.83
	**HMDB0010725**	3-hydroxydecanoic acid	0.2500	0.2575	0.5215	0.5936	0.5392	0.6190	0.5212	0.5352	187.1344	11.31
	**HMDB0010725**	3-hydroxydecanoic acid	0.2467	0.2539	0.5167	0.5869	0.5373	0.6121	0.5140	0.5300	187.1344	11.41
	**HMDB0000193**	Isocitric acid	0.1475	0.1675	0.0832	0.1028	0.0872	0.0873	0.0767	0.0885	191.0203	1.2
	**HMDB0000193**	Isocitric acid	0.0031	0.0037	0.0016	0.0019	0.0018	0.0019	0.0015	0.0015	193.0344	1.11
	**HMDB0000193**	Isocitric acid	0.0289	0.0195	0.0007	0.0011	0.0040	0.0030	0.0006	0.0008	215.0158	1.19
	**HMDB0001149**	5-aminolevulinic acid	0.1225	0.1205	0.1638	0.1555	0.1532	0.1504	0.1719	0.1624	132.0653	0.73
	**HMDB0000112**	4-aminobutanoate (gaba)	0.1152	0.1068	0.2255	0.2118	0.2034	0.2038	0.2370	0.2266	104.0707	0.8
	**HMDB0000112**	4-aminobutanoate (gaba)	0.0107	0.0105	0.0103	0.0100	0.0103	0.0103	0.0101	0.0104	104.0706	0.92
	**HMDB0036458**	1-aminocyclopropane-1-carboxylate	0.1036	0.1041	0.1468	0.1598	0.1407	0.1454	0.1561	0.1587	102.0549	0.74
	**HMDB0000729**	alpha-hydroxyisobutyric acid	0.0965	0.0999	0.1078	0.1082	0.1076	0.1055	0.1120	0.1144	103.0402	2.33
	**HMDB0000070**	Pipecolate/l-pipecolic acid	0.0927	0.0923	0.1188	0.1207	0.1134	0.1078	0.1127	0.1177	130.0862	1.23
	**HMDB0000070**	Pipecolate/l-pipecolic acid	0.0510	0.0521	0.0684	0.0686	0.0635	0.0645	0.0697	0.0702	130.0862	1.32
	**HMDB0000904**	Citrulline	0.0677	0.0670	0.1419	0.1418	0.1287	0.1254	0.1487	0.1508	176.1027	0.78
		alpha-aminoadipate/n-methyl-l-glutamate	0.0548	0.0566	0.0303	0.0339	0.0354	0.0363	0.0294	0.0310	162.0759	0.9
		alpha-aminoadipate/n-methyl-l-glutamate	0.0472	0.0533	0.0482	0.0479	0.0518	0.0455	0.0475	0.0484	162.076	0.78
	**HMDB0059655**	2-hydroxyglutarate	0.0519	0.0560	0.0382	0.0426	0.0424	0.0430	0.0374	0.0383	147.0303	1.51
		2-hydroxyglutarate+na	0.0031	0.0034	0.0022	0.0022	0.0025	0.0025	0.0021	0.0021	171.0266	1.55
		2-hydroxyglutarate-water	0.0212	0.0704	0.1819	0.1820	0.1428	0.1651	0.1937	0.1685	131.0338	1.5
		Alanine/sarcosine	0.0354	0.0360	0.0397	0.0400	0.0404	0.0401	0.0409	0.0401	90.0549	0.9
	**HMDB0000491**	3-methyl-2-oxovaleric acid	0.0345	0.0384	0.0245	0.0255	0.0268	0.0256	0.0244	0.0252	129.056	6.73
	**HMDB0012843**	6-hydroxycaproic acid	0.0292	0.0284	0.0595	0.0568	0.0544	0.0505	0.0718	0.0626	131.0717	7.82
	**HMDB0002064**	N-acetylputrescine	0.0134	0.0131	0.0274	0.0264	0.0238	0.0246	0.0272	0.0253	131.1177	1.13
	**HMDB0011175**	Leu pro	0.0111	0.0211	0.0910	0.1136	0.0777	0.1287	0.0895	0.0876	229.1543	7.17
	**HMDB0011175**	Leu pro	0.0138	0.0212	0.1668	0.1230	0.1228	0.1083	0.1729	0.1189	229.1543	7.36
	**HMDB0000224**	2-aminoethyl dihydrogen phosphate	0.0099	0.0099	0.0073	0.0082	0.0076	0.0082	0.0077	0.0080	140.0122	0.71
	**HMDB0001325**	Nepsilon,nepsilon,nepsilon-trimethyllysine	0.0074	0.0069	0.0085	0.0079	0.0085	0.0084	0.0084	0.0088	189.1595	0.7
	**HMDB0000532**	N-acetylglycine	0.0064	0.0064	0.0154	0.0156	0.0137	0.0142	0.0169	0.0172	116.0354	1.21
	**HMDB0000192**	L-cystine	0.0062	0.0056	0.0087	0.0104	0.0078	0.0090	0.0096	0.0105	241.0306	0.69
	**HMDB0000731**	L-cysteine-s-sulfate	0.0008	0.0009	0.0039	0.0034	0.0029	0.0032	0.0041	0.0039	199.9696	0.73

	**HMDB0062660**	N-methyl-l-glutamate	0.0041	0.0039	0.0020	0.0023	0.0019	0.0022	0.0024	0.0026	160.0618	0.95
	**HMDB0062660**	N-methyl-l-glutamate	0.0040	0.0039	0.0021	0.0022	0.0020	0.0022	0.0023	0.0024	160.0619	0.89
	**HMDB0000026**	3-ureidopropionate	0.0025	0.0029	0.0054	0.0059	0.0037	0.0044	0.0063	0.0064	133.0597	1.43
	**HMDB0003681**	4-acetamidobutanoate	0.0016	0.0016	0.0018	0.0019	0.0017	0.0017	0.0018	0.0019	144.0666	3.56
	**HMDB0003349**	(S)-dihydroorotate	0.0014	0.0018	0.0024	0.0025	0.0024	0.0025	0.0023	0.0022	157.0266	1.14
	**HMDB0000026**	3-ureidopropionate	0.0010	0.0012	0.0009	0.0008	0.0009	0.0010	0.0008	0.0009	133.0604	1.25
	**HMDB0000759**	Glycyl-l-leucine	0.0006	0.0007	0.0033	0.0031	0.0026	0.0025	0.0035	0.0038	189.1231	6.74
	**HMDB0000033**	Carnosine	0.0005	0.0006	0.0006	0.0005	0.0006	0.0003	0.0003	0.0006	227.1138	0.69
	**HMDB0000574**	L-cysteine	0.0001	0.0001	0.0003	0.0005	0.0003	0.0004	0.0004	0.0006	120.0128	0.7
**Lipids and lipid-like molecules**	**HMDB0000086**	Glycerophosphocholine	22.8392	22.1722	18.5881	18.0619	20.0238	19.0859	18.1506	17.5915	258.1096	0.76
		Disaccharide (6c/6c)/2-alpha-D-glucosyl-D-glucose	16.7473	16.3521	16.6134	16.2546	16.3454	16.1464	16.4087	16.6526	377.0867	0.74
		L-carnitine	9.5623	9.5808	8.1115	8.0260	8.6822	8.2668	7.9388	7.9809	162.1122	0.96
	**HMDB0002013**	Butyrylcarnitine	2.0323	2.0082	1.4587	1.4500	1.5658	1.5563	1.3979	1.3675	232.154	7.5
	**HMDB0000824**	Proprionylcarnitine	1.2467	1.2375	0.6482	0.6368	0.8017	0.7913	0.5682	0.5559	218.1384	6.63
	**HMDB0000736**	Isobutyrylcarnitine	0.4593	0.4519	0.2037	0.2002	0.2607	0.2494	0.1713	0.1758	232.154	7.41
	**HMDB0000688**	Isovalerylcarnitine	0.4105	0.4232	0.1586	0.1591	0.2166	0.2218	0.1261	0.1232	246.1696	8.08
	**HMDB0000688**	Isovalerylcarnitine	0.2586	0.2498	0.1070	0.1066	0.1404	0.1403	0.0894	0.0923	246.1696	8.21
	**HMDB0001161**	Deoxycarnitine	0.3792	0.3759	0.3741	0.3733	0.3815	0.3762	0.3788	0.3686	146.1174	1.37
		Glycerol 2-phosphate/SN-glycerol 3-phosphate	0.0730	0.0747	0.0539	0.0539	0.0590	0.0619	0.0519	0.0528	171.0069	0.71
	**HMDB0000201**	Acyl-carnitine(5-OH)	0.0619	0.0620	0.0248	0.0247	0.0343	0.0344	0.0201	0.0200	262.1644	6.6
	**HMDB0000201**	Acyl-carnitine(5-OH)	0.0125	0.0123	0.0042	0.0043	0.0060	0.0060	0.0033	0.0035	262.1645	6.69
	**HMDB0000201**	Acyl-carnitine(5-OH)	0.0063	0.0062	0.0024	0.0024	0.0032	0.0032	0.0019	0.0020	262.1643	6.53
	**HMDB0000126**	SN-glycerol 3-phosphate	0.0550	0.0549	0.0361	0.0374	0.0401	0.0413	0.0357	0.0350	173.0209	0.69
	**HMDB0062477**	2-deoxy-d-galactose (fructose/glucose)	0.0346	0.0372	0.0319	0.0330	0.0329	0.0353	0.0317	0.0316	163.0615	0.91
	**HMDB0000791**	Octanoylcarnitine	0.0295	0.0258	0.0157	0.0146	0.0180	0.0178	0.0142	0.0138	288.2164	10.5
	**HMDB0002833**	Testosterone sulfate	0.0136	0.0131	0.0137	0.0143	0.0144	0.0143	0.0153	0.0148	367.1594	10.73
	**HMDB0000673**	Linoleic acid (18:2)	0.0021	0.0017	0.0014	0.0018	0.0008	0.0013	0.0014	0.0036	279.2335	16.29
	**HMDB0001043**	Arachidonic acid (20:4)	0.0012	0.0007	0.0007	0.0008	0.0006	0.0008	0.0010	0.0023	303.2333	16.04
	**HMDB0006320**	2,6-dimethylheptanoyl carnitine	0.0010	0.0010	0.0006	0.0008	0.0008	0.0008	0.0006	0.0006	302.232	10.71
	**HMDB0000631**	Glycodeoxycholic acid (GDCA)	0.0010	0.0010	0.0006	0.0005	0.0006	0.0006	0.0006	0.0007	448.3083	11.46

	**HMDB0000631**	Glycodeoxycholic acid (GDCA)	0.0002	0.0002	0.0001	0.0001	0.0001	0.0001	0.0001	0.0002	432.3101	11.41
	**HMDB0000138**	Glycocholic acid (GCA)	0.0009	0.0007	0.0013	0.0013	0.0013	0.0010	0.0011	0.0012	464.3028	10.58
	**HMDB0000138**	Glycocholic acid (GCA)	0.0003	0.0003	0.0004	0.0003	0.0003	0.0004	0.0003	0.0003	466.3155	10.55
	**HMDB0000619**	Cholic acid	0.0007	0.0008	0.0007	0.0006	0.0006	0.0006	0.0007	0.0006	373.2735	11.25
**Organic oxygen compounds**	**HMDB0000855**	Nicotinamide	0.2841	0.2058	0.0632	0.0264	0.0997	0.0481	0.0476	0.0237	123.0552	1.46
	**HMDB0000855**	Nicotinamide	0.2788	0.4675	0.5893	0.6259	0.5268	0.6094	0.6125	0.6400	123.0552	1.68
	**HMDB0000855**	Nicotinamide	0.0033	0.0051	0.0063	0.0067	0.0057	0.0063	0.0067	0.0070	121.0417	1.69
	**HMDB0001132**	Nicotinamide mononucleotide	0.0464	0.0490	0.0447	0.0603	0.0387	0.0483	0.0480	0.0543	335.0632	1.09
	**HMDB0000230**	N-acetylneuraminate	0.1032	0.1073	0.1168	0.1224	0.1175	0.1246	0.1182	0.1244	310.1124	0.8
	**HMDB0000230**	N-acetylneuraminate	0.0775	0.0765	0.0814	0.0875	0.0830	0.0855	0.0859	0.0875	308.0996	0.81
	**HMDB0001881**	Dipropylene glycol	0.0783	0.0885	0.0344	0.0323	0.0433	0.0415	0.0245	0.0244	157.0833	6.44
	**HMDB0000684**	L-kynurenine	0.0583	0.0582	0.0567	0.0568	0.0590	0.0570	0.0566	0.0555	209.0917	6.4
	**HMDB0000684**	L-kynurenine	0.0051	0.0052	0.0052	0.0055	0.0052	0.0055	0.0056	0.0054	207.078	6.41
	**HMDB0001129**	N-acetyl-D-mannosamine	0.0463	0.0450	0.0436	0.0449	0.0426	0.0421	0.0454	0.0426	256.0599	0.76
	**HMDB0001367**	N-acetyl-hexosamine	0.0118	0.0101	0.0127	0.0133	0.0123	0.0126	0.0135	0.0134	220.0832	0.82
	**HMDB0001367**	N-acetyl-hexosamine	0.0021	0.0014	0.0024	0.0017	0.0016	0.0017	0.0025	0.0021	244.0787	0.83
	**HMDB0003072**	D-ribose 5-phosphate/ribulose 5-phosphate	0.0092	0.0089	0.0072	0.0062	0.0076	0.0066	0.0070	0.0068	229.0124	0.71
	**HMDB0003072**	Quinate	0.0085	0.0083	0.0077	0.0089	0.0085	0.0080	0.0080	0.0081	191.056	0.79
	**HMDB0060485**	Quinate	0.0034	0.0034	0.0025	0.0023	0.0023	0.0029	0.0017	0.0019	193.0703	0.81
	**HMDB0004086**	Formylkynurenine	0.0063	0.0063	0.0048	0.0047	0.0051	0.0048	0.0046	0.0046	237.0864	10.75
	**HMDB0012948**	5-hydroxy-n-formylkynurenine	0.0057	0.0053	0.0042	0.0038	0.0045	0.0040	0.0038	0.0040	253.0814	11.21
	**HMDB0011631**	Formyl-5-hydroxykynurenamine	0.0044	0.0042	0.0032	0.0030	0.0033	0.0032	0.0030	0.0031	209.0917	11.21
	**HMDB0001473**	3-hydroxykynurenine	0.0033	0.0033	0.0017	0.0015	0.0019	0.0021	0.0013	0.0011	225.0866	3.22
	**HMDB0000618**	Dihydroxyacetone phosphate	0.0027	0.0034	0.0031	0.0028	0.0035	0.0033	0.0030	0.0028	171.0052	0.67
**Organoheterocyclic compounds**	**HMDB0000289**	Ribulose 5-phosphate	0.0020	0.0025	0.0022	0.0020	0.0024	0.0024	0.0022	0.0020	231.0265	0.66
	**HMDB0000289**	Urate	1.8919	1.9254	1.9590	1.9915	1.9350	1.9553	1.9701	2.0069	167.0214	1.88
	**HMDB0002825**	Urate	1.8841	1.9679	1.8565	1.9185	1.9000	1.9208	1.8653	1.8747	169.0353	1.86
		Urate	0.0584	0.0646	0.0546	0.0671	0.0563	0.0816	0.0657	0.0697	167.0211	2.05
		Gluconic acid/D-gulonic acid gama-lactone	0.2015	0.1736	0.0634	0.0570	0.1213	0.0788	0.0563	0.0591	195.0523	0.71

		Theobromine	1.1783	1.1543	0.7718	0.7820	0.8629	0.8514	0.7551	0.7206	181.0717	7.01
	**HMDB0000630**	Theophylline	0.8296	0.8279	0.6008	0.6101	0.6751	0.6519	0.5808	0.5996	181.0716	7.44
	**HMDB0000132**	Isocytosine	0.2464	0.4779	0.4149	0.4935	0.3970	0.5825	0.4246	0.4257	112.0506	1.64
	**HMDB0000132**	Guanine	0.2227	0.2705	0.3071	0.3313	0.2869	0.3826	0.3326	0.3296	152.0564	1.53
	**HMDB0001545**	Guanine	0.0208	0.0289	0.0344	0.0409	0.0314	0.0458	0.0388	0.0392	150.042	1.52
	**HMDB0001545**	Pyridoxal	0.1912	0.1970	0.2254	0.2277	0.2130	0.2079	0.2270	0.2184	168.0653	1.93
	**HMDB0004193**	Pyridoxal	0.0102	0.0106	0.0131	0.0131	0.0120	0.0126	0.0136	0.0144	166.0512	1.95
	**HMDB0062562**	N1-methyl-2-pyridone-5-carboxamide	0.1567	0.1530	0.2146	0.2157	0.1995	0.1944	0.2249	0.2212	153.0656	5.11
	**HMDB0062562**	Urocanate	0.0634	0.0653	0.0638	0.0679	0.0526	0.0626	0.0662	0.1148	139.0501	2.01
	**HMDB0000684**	Urocanate	0.0074	0.0081	0.0076	0.0082	0.0066	0.0072	0.0081	0.0157	137.0357	2.03
	**HMDB0000715**	L-kynurenine	0.0583	0.0582	0.0567	0.0568	0.0590	0.0570	0.0566	0.0555	209.0917	6.4
	**HMDB0000715**	Kynurenic acid	0.0453	0.0467	0.0565	0.0570	0.0544	0.0536	0.0572	0.0589	190.0497	7.78
	**HMDB0060663**	Kynurenic acid	0.0288	0.0294	0.0358	0.0373	0.0357	0.0346	0.0380	0.0392	188.0358	7.79
	**HMDB0003152**	alpha-ketoglutaric acid	0.0415	0.0323	0.0172	0.0133	0.0244	0.0189	0.0141	0.0130	145.0148	0.85
	**HMDB0000076**	N-methylnicotinamide	0.0364	0.0369	0.0365	0.0368	0.0303	0.0366	0.0365	0.0354	137.0708	0.9
	**HMDB0000300**	5,6-dihydrouracil	0.0341	0.0350	0.0277	0.0279	0.0293	0.0299	0.0275	0.0271	115.0501	1.36
	**HMDB0000034**	Uracil	0.0140	0.0106	0.0150	0.0131	0.0152	0.0156	0.0110	0.0160	111.0204	1.34
	**HMDB0000034**	Adenine	0.0128	0.0339	0.0631	0.0562	0.0619	0.0530	0.0572	0.0530	136.0617	1.51
	**HMDB0004077**	Adenine	0.0006	0.0021	0.0040	0.0037	0.0039	0.0036	0.0037	0.0036	134.0479	1.53
	**HMDB0000763**	Dihydroxyquinoine	0.0118	0.0123	0.0126	0.0125	0.0124	0.0121	0.0130	0.0129	162.0548	8
		5-hydroxyindoleacetic acid	0.0106	0.0105	0.0103	0.0101	0.0106	0.0103	0.0101	0.0100	192.0653	6.4
	**HMDB0000017**	Pyridine-2,3-dicarboxylate	0.0059	0.0065	0.0061	0.0060	0.0059	0.0062	0.0057	0.0060	168.0276	1.87
	**HMDB0002894**	4-pyridoxate	0.0054	0.0050	0.0040	0.0038	0.0044	0.0040	0.0038	0.0038	184.0606	5.61
	**HMDB0000763**	5-methylcytosine hydrocloride	0.0046	0.0040	0.0056	0.0056	0.0051	0.0053	0.0058	0.0056	126.0665	1.11
	**HMDB0000881**	5-hydroxyindoleacetic acid	0.0041	0.0043	0.0042	0.0043	0.0043	0.0043	0.0048	0.0046	190.0518	6.4
	**HMDB0000881**	Xanthurenic acid	0.0040	0.0040	0.0022	0.0023	0.0026	0.0026	0.0021	0.0023	204.0308	7.55
	**HMDB0029738**	Xanthurenic acid	0.0040	0.0041	0.0024	0.0024	0.0027	0.0027	0.0022	0.0021	206.0446	7.54
	**HMDB0060484**	Methyl indole-3-acetate	0.0017	0.0016	0.0015	0.0013	0.0014	0.0014	0.0014	0.0014	190.0861	10.63
**Sugars**		Indolepyruvate	0.0005	0.0005	0.0009	0.0008	0.0008	0.0008	0.0010	0.0010	204.0655	6.83
	**HMDB0000186**	Inositol	1.7587	1.9089	1.8656	1.8713	1.9116	1.9107	1.8501	1.8377	179.0565	0.73
		Lactose	1.7509	1.7537	1.6479	1.5358	1.6738	1.5623	1.5900	1.5360	343.1227	0.74

		Lactose+K	0.8829	0.9116	0.8609	0.8911	0.8706	0.8649	0.8437	0.8586	381.0785	0.7
		dimer of lactose	0.1713	0.1746	0.1158	0.1179	0.1283	0.1177	0.1138	0.1186	707.221	0.7
		dimer of lactose	0.0959	0.0895	0.0834	0.0825	0.0841	0.0816	0.0803	0.0786	707.2211	0.79
		Lactate	0.7376	0.8431	0.6484	0.6616	0.6667	0.6793	0.6318	0.7149	89.0242	1.22
		Lactate-dimer	0.0544	0.0577	0.0596	0.0620	0.0598	0.0619	0.0616	0.0650	201.0387	1.22
		C12H22O11-disaccharide-(6c/6c; GLC-GLC/GLC-FRC/GAL-GLC)	1.6094	1.5572	1.6140	1.5865	1.5891	1.5585	1.5930	1.5831	341.1097	0.81
		Glucose/fructose	1.3201	1.3467	1.5755	1.5191	1.4788	1.5015	1.6106	1.5868	215.0334	0.7
		Glucose/fructose	0.4234	0.4354	0.5081	0.4899	0.4765	0.4847	0.5171	0.5089	217.0302	0.7
		Hexose-disaccharide	0.6809	0.7023	0.5138	0.5054	0.5440	0.5234	0.4883	0.4973	365.1046	0.71
	**HMDB0001051**	Hexose-disaccharide	0.4046	0.4070	0.3064	0.2999	0.3112	0.3097	0.2956	0.2925	365.1047	0.76
		Glyceraldehyde	0.5008	0.5692	0.5412	0.5379	0.5600	0.5714	0.5338	0.5344	89.0242	0.74
	**HMDB0000663**	Glucosamine/mannosamine	0.2773	0.2877	0.2682	0.2591	0.2867	0.2540	0.2615	0.2614	180.0864	0.77
		D-saccharic acid	0.1526	0.1946	0.1206	0.1202	0.1256	0.1416	0.1098	0.1048	209.0308	0.72
		Hexose-disaccharide	0.1058	0.0899	0.1082	0.1039	0.0730	0.1126	0.1370	0.1235	365.1047	0.85
		D-glucuronic acid/D-glucuronolactone/D-(+)-galacturonic acid	0.0717	0.0725	0.0638	0.0637	0.0652	0.0616	0.0829	0.0694	193.0358	0.83
		D-glucuronic acid/D-glucuronolactone/D-(+)-galacturonic acid	0.0706	0.0703	0.0648	0.0647	0.0640	0.0604	0.0854	0.0688	193.0358	0.75
	**HMDB0000139**	Aldopentose	0.0650	0.0703	0.0671	0.0674	0.0698	0.0715	0.0692	0.0679	149.0459	0.75
	**HMDB0000849**	Glyceric acid	0.0647	0.0610	0.0845	0.0895	0.0798	0.0828	0.0956	0.1014	105.0194	0.84
	**HMDB0000849**	6-deoxy-hexose	0.0615	0.0615	0.0625	0.0594	0.0610	0.0667	0.0594	0.0562	187.0589	0.89
		6-deoxy-hexose	0.0212	0.0227	0.0245	0.0205	0.0252	0.0184	0.0197	0.0261	187.0592	0.82
	**HMDB0000765**	Mannitol/sorbitol	0.0186	0.0199	0.0228	0.0216	0.0215	0.0208	0.0240	0.0224	183.0863	0.73
**Nucleosides, nucleotides, and analogues**	**HMDB0000089**	Mannitol	0.0098	0.0092	0.0114	0.0118	0.0110	0.0102	0.0114	0.0113	181.0719	0.73
	**HMDB0000089**	Cytidine	0.1965	0.3846	0.3336	0.3954	0.3232	0.4594	0.3426	0.3483	244.092	1.64
	**HMDB0000050**	Cytidine	0.0631	0.1295	0.1124	0.1380	0.1070	0.1446	0.1188	0.1245	242.0787	1.66
	**HMDB0000296**	Adenosine	0.1695	0.1491	0.0589	0.0594	0.0889	0.0718	0.0456	0.0481	268.1035	5.73
	**HMDB0000288**	Uridine	0.0909	0.1364	0.2588	0.2886	0.2313	0.2576	0.2737	0.3042	243.0626	2.87
	**HMDB0000095**	Uridine-5-monophosphate	0.0443	0.0389	0.0376	0.0351	0.0421	0.0393	0.0382	0.0385	323.0293	1.24
	**HMDB0000095**	CMP	0.0421	0.0506	0.0598	0.0660	0.0608	0.0613	0.0639	0.0717	324.0585	1.04
	**HMDB0240312**	CMP	0.0289	0.0335	0.0500	0.0577	0.0489	0.0523	0.0578	0.0626	322.0452	1.05

	**HMDB0240312**	3'-CMP	0.0040	0.0056	0.0100	0.0192	0.0095	0.0133	0.0124	0.0147	322.0452	1.53
	**HMDB0240312**	3'-CMP	0.0031	0.0059	0.0047	0.0073	0.0050	0.0067	0.0049	0.0062	322.0451	1.59
	**HMDB0240312**	3'-CMP	0.0024	0.0034	0.0056	0.0106	0.0054	0.0077	0.0069	0.0083	324.0585	1.52
	**HMDB0000229**	3'-CMP	0.0007	0.0009	0.0014	0.0031	0.0014	0.0023	0.0021	0.0021	324.0585	1.75
	**HMDB0000045**	Nicotinamide ribotide	0.0279	0.0273	0.0309	0.0407	0.0274	0.0322	0.0355	0.0352	379.0558	1.11
	**HMDB0000045**	Adenosine 5'-monophosphate	0.0271	0.0204	0.0071	0.0048	0.0115	0.0069	0.0050	0.0039	348.0697	1.66
	**HMDB0000296**	Adenosine 5'-monophosphate	0.0267	0.0192	0.0070	0.0053	0.0111	0.0070	0.0052	0.0044	346.0566	1.68
	**HMDB0000288**	Uridine	0.0236	0.0340	0.0675	0.0752	0.0594	0.0685	0.0744	0.0785	267.0583	2.85
	**HMDB0001517**	Uridine-5-monophosphate	0.0166	0.0140	0.0119	0.0112	0.0144	0.0127	0.0120	0.0115	325.0425	1.22
	**HMDB0001517**	5-aminoimidazole-4-carboxamide-1-beta-d-ribofuranosyl 5'-monophosphate	0.0066	0.0069	0.0154	0.0187	0.0163	0.0179	0.0173	0.0194	337.0558	1.46
	**HMDB0000133**	5-aminoimidazole-4-carboxamide-1-beta-d-ribofuranosyl 5'-monophosphate	0.0055	0.0053	0.0110	0.0134	0.0123	0.0126	0.0123	0.0134	339.0695	1.46
	**HMDB0001859**	Guanosine	0.0022	0.0028	0.0029	0.0032	0.0025	0.0032	0.0029	0.0028	284.0984	5.98
**Benzenoids**	**HMDB0000714**	Acetaminophen/paracetamol	0.8834	0.9295	0.4742	0.4739	0.6127	0.6112	0.4201	0.4071	152.0705	7.06
	**HMDB0000840**	Hippuric acid	0.5775	0.6040	0.6228	0.6228	0.6123	0.5940	0.6371	0.6340	180.0653	8
	**HMDB0000840**	2-hydroxyhippuric acid	0.0306	0.0305	0.0211	0.0219	0.0238	0.0238	0.0204	0.0204	194.0463	7.46
	**HMDB0006116**	2-hydroxyhippuric acid	0.0194	0.0197	0.0129	0.0107	0.0120	0.0118	0.0100	0.0119	194.0463	7.28
	**HMDB0060026**	Hydroxyhippuric acid	0.0301	0.0312	0.0309	0.0297	0.0303	0.0298	0.0310	0.0301	196.0392	8
	**HMDB0001123**	Vanilloylglycine	0.0070	0.0076	0.0119	0.0126	0.0109	0.0118	0.0131	0.0125	224.0572	7.71
**Organic nitrogen compounds**	**HMDB0001565**	Anthranilate	0.0060	0.0060	0.0055	0.0054	0.0055	0.0054	0.0054	0.0053	120.0443	8.7
	**HMDB0001565**	Phosphocholine	10.8029	10.4934	6.5314	7.0740	7.8247	7.6993	6.4746	6.3702	184.0732	0.72
	**HMDB0001414**	Phosphocholine	0.0848	0.0828	0.0689	0.0694	0.0722	0.0739	0.0671	0.0656	218.0341	0.71
	**HMDB0001861**	Putrescine	0.0056	0.0063	0.0081	0.0090	0.0081	0.0078	0.0084	0.0085	89.1073	0.59
**Vitamins**	**HMDB0000097**	3-methylhistamine	0.0016	0.0018	0.0025	0.0022	0.0021	0.0025	0.0024	0.0027	126.1025	0.69
	**HMDB0000210**	Choline	14.8723	16.6452	15.1259	14.8088	14.9679	14.6185	15.1332	14.8174	104.1069	0.83
	**HMDB0000210**	Pantothenic acid	1.6457	1.6466	2.1475	2.1542	2.0539	2.0038	2.2017	2.1464	220.1176	6.55
		Pantothenic acid	0.9460	0.9865	1.2538	1.2986	1.2132	1.2102	1.3170	1.3206	218.1036	6.54
		Ascorbic acid-2H	0.9981	0.7384	0.2309	0.1644	0.4417	0.3057	0.1634	0.1307	173.0094	1.03

		Ascorbic acid-2H	0.9981	0.7384	0.2620	0.1753	0.4535	0.3166	0.1653	0.1287	173.0094	0.87
	**HMDB0000044**	Ascorbic acid-2H	0.9813	0.7316	0.2574	0.1704	0.4403	0.3116	0.1640	0.1309	173.0094	0.96
		Ascorbic acid	0.8418	0.5525	0.0273	0.0323	0.0230	0.0266	0.0285	0.0320	175.0252	0.87
**Standards and Others**		5-hydroxymethyl-2-furaldehyde	3.1771	3.2714	2.9595	2.8184	2.9951	2.9279	2.8964	2.8255	127.0389	0.75
		BOC-L-tyrosine	1.9871	1.9159	1.5831	1.4552	1.6615	1.5757	1.5516	1.4309	182.081	10.09
		BOC-L-tyrosine	0.8752	0.8608	0.7774	0.7442	0.7911	0.7471	0.7740	0.7472	280.1196	10.1
		BOC-L-tyrosine	0.0320	0.0313	0.0355	0.0373	0.0331	0.0341	0.0379	0.0378	304.1149	10.08
		L-tyrosine-13C6	0.0235	0.0235	0.0223	0.0232	0.0232	0.0222	0.0232	0.0241	186.087	3.42
		BOC-D-phenylalanine	1.9641	1.9018	1.4260	1.3162	1.4495	1.4071	1.3963	1.3648	166.0861	11.49
		BOC-D-phenylalanine	0.0112	0.0108	0.0086	0.0076	0.0086	0.0081	0.0080	0.0081	288.1198	11.49
		L-phenylalanine 13C6	1.0160	0.9976	0.9242	0.9198	0.9421	0.9385	0.9292	0.9204	172.1062	6.17
		L-phenylalanine-13C6	0.0289	0.0274	0.0261	0.0263	0.0265	0.0254	0.0269	0.0266	170.0922	6.16
		N-BOC-L-tryptophan	1.3378	1.3358	0.9499	0.9033	0.9790	0.9438	0.9629	0.8923	205.0969	11.52
		N-BOC-L-tryptophan	1.1348	1.1427	0.9210	0.8991	0.9280	0.9220	0.9332	0.9159	229.0623	11.54
		N-BOC-L-tryptophan	0.8243	0.8170	0.6652	0.6508	0.6799	0.6529	0.6845	0.6570	303.1357	11.54
		N-BOC-L-tryptophan	0.1397	0.1386	0.0931	0.0898	0.0983	0.0937	0.0964	0.0879	327.1307	11.52
		Tryptophan-2,3,3-D3-NH3	3.3329	3.3112	3.0188	3.0770	3.1295	3.0749	3.1259	3.0111	191.0892	7.71
		Tryptophan-2,3,3-D3	0.7709	0.7549	0.7266	0.7439	0.7318	0.7183	0.7468	0.7457	206.1018	7.71
		N-BOC-L-aspartic acid	0.7733	0.7809	0.7355	0.7571	0.7497	0.7302	0.7498	0.7406	232.0831	8.59
		N-BOC-L-aspartic acid	0.3209	0.3164	0.2750	0.2772	0.2830	0.2843	0.2707	0.2641	134.0447	8.59
		N-BOC-L-aspartic acid	0.1596	0.1610	0.1512	0.1563	0.1526	0.1543	0.1534	0.1521	158.0098	8.59
		N-BOC-L-tert-leucine	0.7081	0.7000	0.6080	0.6218	0.6120	0.6105	0.6191	0.6153	156.067	10.96
		N-BOC-L-aspartic acid	0.0180	0.0173	0.0149	0.0152	0.0154	0.0161	0.0150	0.0142	256.0786	8.59
		4-oxoproline	0.2421	0.2826	0.7972	0.7969	0.6770	0.7153	0.8313	0.9148	128.0356	1.84
		N-BOC-L-tert-leucine	0.2277	0.2220	0.1965	0.1987	0.2000	0.1893	0.2051	0.1987	230.1402	10.96
		N-BOC-L-tert-leucine	0.1813	0.1754	0.1499	0.1474	0.1532	0.1485	0.1480	0.1437	132.1017	10.95
		Succinic-2,2,3,3-D4 acid	0.1931	0.1948	0.1848	0.1884	0.1877	0.1890	0.1842	0.1869	121.0447	2.27
		Salicylic acid-D4	0.1283	0.1288	0.1112	0.1136	0.1100	0.1086	0.1125	0.1123	141.0499	10.04
		4-hydroxybenzenesulfonic acid	0.0991	0.1002	0.0460	0.0472	0.0578	0.0553	0.0410	0.0443	172.9916	7.14
		Trigonelline	0.0745	0.0801	0.0733	0.0830	0.0792	0.0816	0.0845	0.0879	138.0545	0.85
		Trigonelline	0.0696	0.0764	0.0695	0.0785	0.0730	0.0731	0.0680	0.0736	138.0548	0.94
		Orthophosphate	0.0578	0.0589	0.0212	0.0239	0.0231	0.0247	0.0239	0.0223	98.9839	0.71

		4-hydroxy-l-phenylglycine	0.0446	0.0486	0.0458	0.0460	0.0461	0.0460	0.0449	0.0461	168.0652	0.8
		3,4-dihydroxybenzenesulfonic acid	0.0161	0.0158	0.0243	0.0247	0.0223	0.0223	0.0246	0.0258	188.9867	6.67
		Creatine-D3	0.6861	0.6658	0.5976	0.6017	0.6310	0.6159	0.5835	0.5816	135.0955	0.9
		Creatine-D3	0.0018	0.0015	0.0020	0.0021	0.0016	0.0018	0.0026	0.0025	133.0816	0.91
		L-leucine 13C6	1.2771	1.2353	1.1098	1.1091	1.1100	1.1499	1.0785	1.0942	138.1219	2.53
		L-leucine-13C6	0.0020	0.0019	0.0016	0.0018	0.0018	0.0018	0.0017	0.0017	136.1079	2.53
		L-leucine-D10	0.7790	0.7757	0.7312	0.7367	0.7167	0.7550	0.7242	0.7323	142.1646	2.49
		Tryptophan-2,3,3-D3	7.7381	7.7476	7.0890	7.1740	7.2828	7.1451	7.2684	7.0514	208.1159	7.71
		Caffeine-D3	1.1508	1.1220	1.0206	1.0245	1.0535	1.0275	1.0288	1.0161	198.1061	7.99
		7-(b-hydroxyethyl) theophylline	0.0071	0.0074	0.0107	0.0106	0.0092	0.0096	0.0106	0.0107	225.0981	7.83

	647.3489	8.52	Unknown	-	3.39	0.0174
	650.3626	8.43	Unknown	-	2.46	0.0028
	738.9095	8.82	Unknown	-	2.83	0.0047
	836.4768	10.23	Unknown	-	3.06	0.0217
	924.2429	10.09	Unknown	-	2.36	0.0203
**Negative ionization mode**	138.0566	7.78	Unknown	-	3.56	0.0377
	166.0375	1.34	Unknown	-	5.62	0.0144
	178.0372	3.71	Unknown	-	32.07	0.0064
	182.047	7.73	Unknown	-	5.31	0.0494
	182.0473	7.81	Unknown	-	15.47	0.0115
	185.1184	10.75	Unknown	-	3.16	0.0005
	197.0318	3.29	Unknown	-	2.21	0.0193
	204.0703	7.79	Unknown	-	3.05	0.0031
	229.0217	1.88	Unknown	-	2.53	0.0347
	235.0342	1.69	Unknown	-	3.51	0.0098
	240.0229	3.42	Unknown	-	2.77	0.0356
	256.1249	11.41	Unknown	-	2.69	0.0320
	257.0898	3.19	Unknown	-	6.55	0.0030
	272.1508	10.32	Unknown	-	8.77	0.0062
	272.9933	1.24	Unknown	-	3.06	0.0101
	280.99	1.31	Unknown	-	3.51	0.0145
	304.0792	1.66	Unknown	-	3.29	0.0446
	317.1147	8.91	Unknown	-	100.00	0.0237

	322.0452	1.53	Unknown	-	19.54	0.0296
	322.0452	1.05	Unknown	-	3.57	0.0497
	335.0646	1.1	Unknown	-	3.63	0.0174
	348.0598	8.21	Unknown	-	2.41	0.0465
	357.056	2.87	Unknown	-	2.82	0.0184
	379.0558	1.11	Unknown	-	5.10	0.0005
	383.123	3.42	Unknown	-	4.31	0.0040
	565.0247	1.9	Unknown	-	28.47	0.0027

The compounds are grouped by ionization mode. Feature number corresponds to a unique identification obtained from MZmine. The ID codes correspond to Human Metabolome Database.

Table S5. Concentration of glutamine and choline derivative forms.

Sample	Choline	Glycerophosphocholine	Phosphocholine	Glutamine
MOM T0	14.9 ±6.0	22.8 ±9.3	10.9 ±6.2	3.1 ±2.2
MOM T4	16.6 ±7.5	22.2 ±8.9	10.6 ±6.3	2.7 ±1.9
RM10 T0	15.1 ±2.1	18.6 ±3.4	6.6 ±2.3	5.9 ±4.3
RM10 T4	14.8 ±1.8	18.1 ±4.0	7.1 ±2.5	5.9 ±4.3
RM30 T0	15.0 ±2.3	20.0 ±4.9	7.9 ±2.9	5.5 ±3.6
RM30 T4	14.6 ±1.9	19.1 ±4.5	7.8 ±2.4	5.4 ±3.7
DHM T0	15.1 ±2.3	18.2 ±2.5	6.5 ±2.4	6.1 ±4.7
DHM T4	14.8 ±2.5	17.6 ±3.4	6.4 ±2.2	6.2 ±4.7

Relative percentage of water-soluble choline forms are listed as (Mean±SD). T-test was performed to compare the means, no significant differences were found ($p > 0.05$).

Table S6. Molecules Regulation and Top Functions Implicated

Network	Analysis	Molecules in Network	Score	Focus molecules	Top Functions
1	MOM T0	**5-hydroxyindol-3-acetic acid**↓, ACAC, **acetyl-L-carnitine**↑, Aconitase, arginase, **carnosine**↑, **citric acid**↑, **citrulline**↓, Collagen Alpha 1, creatine kinase, **delta-aminolevulinic acid**↓, ERK1/2, Ferritin, glutathione peroxidase, **guanosine**↓, **kynurenic acid**↓, **L-alanine**↓, **L-arginine**↓, **L-aspartic acid**↑, **L-carnitine**↑, **L-cystine**↓, **L-glutamine**↓, **L-histidine**↓, **L-kynurenine**↑, **L-Leucine**↓, **L-phenylalanine**↓, **L-proline**↑, **lactose**↑, Ldh(complex), malate dehydrogenase, **methionine sulfoxide**↓, **phosphate**↑, proinsuline, Sod, **uric acid**↓	49	23	Cellular Compromise, Lipid Metabolism, Small Molecule Biochemistry

	MOM T4	**5-hydroxyindol-3-acetic acid**↓, ACAC, **acetyl-L-carnitine**↑, ALT, Amylase, arginase, **carnosine**↑, **citric acid**↑, **citrulline**↓, creatine kinase, **creatinine**↑, **delta-aminolevulinic acid**↓, ERK1/2, Ferritin, **GABA**↓, glutathione peroxidase, **glycine**↓, Glycine receptor, GOT, **guanosine**↓, **kynurenic acid**↓, **L-alanine**↓, **L-arginine**↓, **L-carnitine**↑, **L-cystine**↓, **L-glutamine**↓, **L-kynurenine**↑, **L-proline**↑, **lactose**↑, Ldh(complex), malate dehydrogenase, **methionine sulfoxide**↓, **phosphate**↑, Sod, **taurine**↓	47	22
	RM10 T0	**5-hydroxyindol-3-acetic acid**↓, ACAC, **acetyl-L-carnitine**↑, Aconitase, arginase, **carnosine**↑, **citric acid**↓, **citrulline**↓, Collagen Alpha 1, creatine kinase, **delta-aminolevulinic acid**↓, ERK1/2, Ferritin, glutathione peroxidase, **guanosine**↓, **kynurenic acid**↓, **L-alanine**↓, **L-arginine**↓, **L-aspartic acid**↓, **L-carnitine**↑, **L-cystine**↓, **L-glutamine**↓, **L-histidine**↓, **L-kynurenine**↓, **L-Leucine**↑, **L-phenylalanine**↑, **L-proline**↓, **lactose**↑, Ldh(complex), malate dehydrogenase, **methionine sulfoxide**↓, **phosphate**↓, proinsuline, Sod, **uric acid**↓	49	23
	RM10 T4	**5-hydroxyindol-3-acetic acid**↓, ACAC, **acetyl-L-carnitine**↑, Aconitase, arginase, **carnosine**↑, **citric acid**↑, **citrulline**↓, Collagen Alpha 1, creatine kinase, **delta-aminolevulinic acid**↓, ERK1/2, Ferritin, glutathione peroxidase, **guanosine**↑, **kynurenic acid**↓, **L-alanine**↓, **L-arginine**↑, **L-aspartic acid**↑, **L-carnitine**↓, **L-cystine**↑, **L-glutamine**↓, **L-histidine**↓, **L-kynurenine**↑, **L-Leucine**↑, **L-phenylalanine**↑, **L-proline**↑, **lactose**↓, Ldh(complex), malate dehydrogenase, **methionine sulfoxide**↓, **phosphate**↓, proinsuline, Sod, **uric acid**↑	49	23
	RM30 T0	**5-hydroxyindol-3-acetic acid**↑, ACAC, **acetyl-L-carnitine**↑, Aconitase, arginase, **carnosine**↑, **citric acid**↑, **citrulline**↓, Collagen Alpha 1, creatine kinase, **delta-aminolevulinic acid**↓, ERK1/2, Ferritin, glutathione peroxidase, **guanosine**↓, **kynurenic acid**↓, **L-alanine**↓, **L-arginine**↓, **L-aspartic acid**↓, **L-carnitine**↑, **L-cystine**↓, **L-glutamine**↓, **L-histidine**↓, **L-kynurenine**↑, **L-Leucine**↓, **L-phenylalanine**↓, **L-proline**↑, **lactose**↑, Ldh(complex), malate dehydrogenase, **methionine sulfoxide**↓, **phosphate**↓, proinsuline, Sod, **uric acid**↓	49	23
	RM30 T4	**5-hydroxyindol-3-acetic acid**↓, ACAC, **acetyl-L-carnitine**↑, Aconitase, arginase, **carnosine**↑, **citric acid**↑, **citrulline**↓, Collagen Alpha 1, creatine kinase, **delta-aminolevulinic acid**↓, ERK1/2, Ferritin, glutathione peroxidase, **guanosine**↑, **kynurenic acid**↓, **L-alanine**↓, **L-arginine**↑, **L-aspartic acid**↑, **L-carnitine**↑, **L-cystine**↓, **L-glutamine**↓, **L-histidine**↑, **L-kynurenine**↑, **L-Leucine**↑, **L-phenylalanine**↑, **L-proline**↑, **lactose**↓, Ldh(complex), malate dehydrogenase, **methionine sulfoxide**↑, **phosphate**↑, proinsuline, Sod, **uric acid**↑	49	23
	DHM T4	**5-hydroxyindol-3-acetic acid**↓, ACAC, **acetyl-L-carnitine**↑, Aconitase, arginase, **carnosine**↑, **citric acid**↑, **citrulline**↑, Collagen Alpha 1, creatine kinase, **delta-aminolevulinic acid**↓, ERK1/2, Ferritin, glutathione peroxidase, **guanosine**↓, **kynurenic acid**↑, **L-alanine**↓, **L-arginine**↑, **L-aspartic acid**↑, **L-carnitine**↓, **L-cystine**↑, **L-glutamine**↑, **L-histidine**↑, **L-kynurenine**↓, **L-Leucine**↑, **L-phenylalanine**↑, **L-proline**↑, **lactose**↓, Ldh(complex), malate dehydrogenase, **methionine sulfoxide**↓, **phosphate**↓, proinsuline, Sod, **uric acid**↑	49	23

2	MOM T0	Akt, ALT, amylase, **choline**↓, **creatinine**↑, CYP, **cytosine**↓, **GABA**↓, Ggt, **glucosamine**↑, glucose transporter, **glycine**↓, Glycine receptor, **L-asparagine**↓, **L-cysteine**↓, **L-glutamic acid**↓, **L-isoleucine**↓, **L-lactic acid**↑, **L-lysine**↓, **L-methionine**↑, **L-threonine**↓, L-type Calcium channel, **L-valine**↑, **malic acid**↑, **mannitol**↓, N-type calcium channel, nicotinic acetylcholine receptor, 70 S6k, **phosphorylcholine**↑, **sorbitol**↓, succinate dehydrogenase, **succinic acid**↓, **taurine**↓, transglutaminase, vacuolar H ATPase	44	21	Cellular growth and proliferation
	MOM T4	Akt, **AMP**↑, AMPK, **ascorbic acid**↑, **choline**↑, Ggt, **glucosamine**↑, glucose transporter, **L-asparagine**↓, **L-aspartic acid**↑, **L-cysteine**↓, **L-histidine**↓, **L-isoleucine**↑, **L-luecine**↓, **L-lysine**↓, **L-methionine**↑, **L-phenylalanine**↓, **L-serine**↓, **L-threonine**↓, **L-tryptophan**↓, **L-valine**↑, **mannitol**↓, MTORC1, NADPH oxidase, 70 S6k, Pld, PP2A, PRKAA, Proinsulin, pyruvate kinase, **sorbitol**↓, succinate dehydrogenase, **succinic acid**↓, transglutaminase, vacuolar H ATPase	41	20	
	RM10 T0	Akt, ALT, amylase, **choline**↓, **creatinine**↑, CYP, **cytosine**↓, **GABA**↓, Ggt, **glucosamine**↑, glucose transporter, **glycine**↓, Glycine receptor, **L-asparagine**↓, **L-cysteine**↓, **L-glutamic acid**↓, **L-isoleucine**↑, **L-lactic acid**↑, **L-lysine**↓, **L-methionine**↑, **L-threonine**↓, L-type Calcium channel, **L-valine**↑, **malic acid**↑, **mannitol**↑, N-type calcium channel, nicotinic acetylcholine receptor, 70 S6k, **phosphorylcholine**↑, **sorbitol**↓, succinate dehydrogenase, **succinic acid**↑, **taurine**↓, transglutaminase, vacuolar H ATPase	44	21	
	RM10 T4	Akt, ALT, amylase, **choline**↓, **creatinine**↓, CYP, **cytosine**↑, **GABA**↓, Ggt, **glucosamine**↓, glucose transporter, **glycine**↓, Glycine receptor, **L-asparagine**↓, **L-cysteine**↑, **L-glutamic acid**↑, **L-isoleucine**↑, **L-lactic acid**↓, **L-lysine**↑, **L-methionine**↑, **L-threonine**↓, L-type Calcium channel, **L-valine**↑, **malic acid**↑, **mannitol**↑, N-type calcium channel, nicotinic acetylcholine receptor, 70 S6k, **phosphorylcholine**↑, **sorbitol**↓, succinate dehydrogenase, **succinic acid**↑, **taurine**↓, transglutaminase, vacuolar H ATPase	44	21	
	RM30 T0	Akt, ALT, amylase, **choline**↓, **creatinine**↑, CYP, **cytosine**↓, **GABA**↓, Ggt, **glucosamine**↑, glucose transporter, **glycine**↓, Glycine receptor, **L-asparagine**↓, **L-cysteine**↓, **L-glutamic acid**↓, **L-isoleucine**↑, **L-lactic acid**↑, **L-lysine**↓, **L-methionine**↓, **L-threonine**↓, L-type Calcium channel, **L-valine**↑, **malic acid**↑, **mannitol**↓, N-type calcium channel, nicotinic acetylcholine receptor, 70 S6k, **phosphorylcholine**↑, **sorbitol**↓, succinate dehydrogenase, **succinic acid**↓, **taurine**↓, transglutaminase, vacuolar H ATPase	44	21	
	RM30 T4	Akt, ALT, amylase, **choline**↓, **creatinine**↓, CYP, **cytosine**↑, **GABA**↓, Ggt, **glucosamine**↓, glucose transporter, **glycine**↑, Glycine receptor, **L-asparagine**↓, **L-cysteine**↓, **L-glutamic acid**↓, **L-isoleucine**↑, **L-lactic acid**↑, **L-lysine**↓, **L-methionine**↑, **L-threonine**↓, L-type Calcium channel, **L-valine**↑, **malic acid**↑, **mannitol**↓, N-type calcium channel, nicotinic acetylcholine receptor, 70 S6k, **phosphorylcholine**↑, **sorbitol**↓, succinate dehydrogenase, **succinic acid**↑, **taurine**↓, transglutaminase, vacuolar H ATPase	44	21	

	DHM T4	Akt, ALT, amylase, **choline**↓, **creatinine**↓, CYP, **cytosine**↑, **GABA**↓, Ggt, **glucosamine**↓, glucose transporter, **glycine**↓, Glycine receptor, **L-asparagine**↓, **L-cysteine**↑, **L-glutamic acid**↑, **L-isoleucine**↑, **L-lactic acid**↑, **L-lysine**↑, **L-methionine**↑, **L-threonine**↓, L-type Calcium channel, **L-valine**↑, **malic acid**↓, **mannitol**↓, N-type calcium channel, nicotinic acetylcholine receptor, 70 S6k, **phosphorylcholine**↓, **sorbitol**↓, succinate dehydrogenase, **succinic acid**↑, **taurine**↓, transglutaminase, vacuolar H ATPase	44	21	
3	MOM T0	**5'-CMP**↓, 8-epi-prostaglandin F2alpha, ALB, **alpha-keto-beta-methylvaleric acid**↑, **ascorbic acid**↑, BCL2L1, CASP10, **citrulline**↓, copper, **D-pantothenic acid**↓, DPP4, EGFR, FAH, **glycylleucine**↓, **hippuric acid**↓, HTT, **kyrurenic acid**↓, **L-aspartic acid**↑, **L-lysine**↓, **myo-inositol**↓, N-acetyl-L-aspartic acid, **N-acetyl-L-serine**↑, PACSIN2, **phosphorylcholine**↑, **phosphorylethanolamine**↑, **putrescine**↓, SLC22A6, transglutaminase, UDP-D-glucose, UDP-N-acetylglucosamine, **uracil**↑, **uridine**↓, **xanthurenic acid**↑	36	18	Inflammatory Response
	MOM T4	**5'-CMP**↓, 8-epi-prostaglandin F2alpha, ALB, **alpha-keto-beta-methylvaleric acid**↑, **ascorbic acid**↑, BCL2L1, CASP10, **citrulline**↓, copper, **D-pantothenic acid**↓, DPP4, EGFR, FAH, **glycylleucine**↓, **hippuric acid**↓, HTT, **kyrurenic acid**↓, **L-aspartic acid**↑, **L-carnitine**↑, **L-cysteine**↓, **myo-inositol**↑, N-acetyl-L-aspartic acid, **N-acetyl-L-serine**↓, PACSIN2, phosphocreatine, **phosphorylcholine**↑, **phosphorylethanolamine**↑, **putrescine**↓, SLC22A6, transglutaminase, UDP-D-glucose, UDP-N-acetylglucosamine, **uracil**↓, **uridine**↓, **xanthurenic acid**↑	38	19	
	RM10 T0	**5'-CMP**↓, 8-epi-prostaglandin F2alpha, ALB, **alpha-keto-beta-methylvaleric acid**↑, alpha-ketoisocaproic acid, BCL2L1, **citrulline**↓, **D-pantothenic acid**↓, DPP4, EGFR, FAH, **glycylleucine**↓, **hippuric acid**↓, HTT, **kyrurenic acid**↓, **L-aspartic acid**↓, **L-carnitine**↑, **L-cysteine**↓, **L-phenylalaninel**↑, **L-proline**↓, **myo-inositol**↑, N-acetyl-L-aspartic acid, **N-acetyl-L-serine**↑, phosphocreatine, **phosphorylcholine**↑, **phosphorylethanolamine**↓, **putrescine**↓, SLC22A6, UDP-D-glucose, UDP-N-acetylglucosamine, **uracil**↑, **uridine**↓, **xanthurenic acid**↑	44	21	
	RM10 T4	**5'-CMP**↑, 8-epi-prostaglandin F2alpha, ALB, **alpha-keto-beta-methylvaleric acid**↑, alpha-ketoisocaproic acid, BCL2L1, **citrulline**↓, CYP2J2, **D-pantothenic acid**↓, DPP4, EGFR, FAH, **glycylleucine**↓, **hippuric acid**↓, HTT, **kyrurenic acid**↓, **L-aspartic acid**↑, **L-carnitine**↓, **L-cysteine**↑, **L-phenylalanine**↑, **L-proline**↑, **myo-inositol**↑, N-acetyl-L-aspartic acid, **N-acetyl-L-serine**↑, phosphocreatine, **phosphorylcholine**↑, **phosphorylethanolamine**↑, **putrescine**↑, SLC22A6, transglutaminase, UDP-D-glucose, UDP-N-acetylglucosamine, **uracil**↑, **uridine**↑, **xanthurenic acid**↑	44	21	
	RM30 T0	**5'-CMP**↓, 8-epi-prostaglandin F2alpha, ALB, **alpha-keto-beta-methylvaleric acid**↑, alpha-ketoisocaproic acid, **ascorbic acid**↑, BCL2L1, **citrulline**↓, copper, **D-pantothenic acid**↓, DPP4, EGFR, FAH, **glycine**↓, **glycylleucine**↓, **hippuric acid**↓, HTT, **kyrurenic acid**↓, **L-aspartic acid**↓, **L-carnitine**↑, **L-cysteine**↓, **myo-inositol**↑, N-acetyl-L-aspartic acid, **N-acetyl-L-serine**↑, phosphocreatine, **phosphorylcholine**↑, **phosphorylethanolamine**↓, **putrescine**↓, SLC22A6, UDP-D-glucose, UDP-N-acetylglucosamine, **uracil**↑, **uridine**↓, **xanthurenic acid**↑	41	20	

	RM30 T4	**5'-CMP**↓, 8-epi-prostaglandin F2alpha, ALB, **alpha-keto-beta-methylvaleric acid**↑, alpha-ketoisocaproic acid, BCL2L1, **citrulline**↓, **D-pantothenic acid**↓, DPP4, EGFR, FAH, **glycine**↑, **glycylleucine**↓, **hippuric acid**↓, HTT, **kyrurenic acid**↓, **L-aspartic acid**↑, **L-carnitine**↑, **L-cysteine**↓, **L-phenylalanine**↑, **L-proline**↑, **myo-inositol**↑, N-acetyl-L-aspartic acid, **N-acetyl-L-serine**↑, phosphocreatine, **phosphorylcholine**↑, **phosphorylethanolamine**↑, **putrescine**↓, SLC22A6, UDP-D-glucose, UDP-N-acetylglucosamine, **uracil**↑, **uridine**↓, **xanthurenic acid**↑	44	21	
	DHM T4	**5'-CMP**↑, ALB, **alpha-keto-beta-methylvaleric acid**↑, alpha-ketoisocaproic acid, BCL2L1, **citrulline**↑, **D-pantothenic acid**↓, Dgk, DPP4, EGFR, FAH, **glycine**↓, **glycylleucine**↑, **hippuric acid**↓, HTT, **kyrurenic acid**↑, **L-aspartic acid**↑, **L-carnitine**↓, **L-cysteine**↑, **myo-inositol**↓, N-acetyl-L-aspartic acid, **N-acetyl-L-serine**↑, palmitoleic acid, phosphocreatine, **phosphorylcholine**↓, **phosphorylethanolamine**↑, **putrescine**↑, SLC22A6, UDP-D-glucose, UDP-N-acetylglucosamine, **uracil**↑, **uridine**↑, **xanthurenic acid**↑	38	19	
4	MOM T0	**1-aminocyclopropane-1-carboxylic acid**↓, 8-epi-prostaglandin F2alpha, **aceturic acid**↓, **adenine**↓, AGT, **beta-lactose**↑, **D-gluconic acid**↑, **D-ribose-5-phosphate**↑, **dihydrouracil**↑, ethanol, Glycine receptor, **glycocholic acid**↓, **glycocyamine**↑, **glycoursodeoxycholic acid**↑, GRIN2B, GRIN3A, GRIN3B, GUSb, IL1B, IL37, ion channel, LOLRA3, MMP9, **N'-methyl-2-pyridone-5-carboxamide**↓, **N-acetylneuraminic acid**↓, NAD, **nicotinamide-beta-riboside**↓, nitrate, PARP1, PPARGC1A, RPTOR, **sorcosine**↓, SLCO1B1, thromboxane B2, **trigonelline**↓	28	15	Nucleic acid metabolism, Amino Acid Metabolism, carbohydrate metabolism, molecular transport
	MOM T4	**1-aminocyclopropane-1-carboxylic acid**↓, 8-epi-prostaglandin F2alpha, **aceturic acid**↓, **beta-lactose**↑, **citrulline**↓, **D-gluconic acid**↑, **D-ribose-5-phosphate**↑, **dihydrouracil**↑, ethanol, Glycine receptor, **glycocyamine**↑, GRIN1, GRIN3A, GRIN3B, hydroxyproline, LILRA3, MAS1, MMP9, **N'-methyl-2-pyridone-5-carboxamide**↓, **N-acetylneuraminic acid**↓, nicotamide adenine dinucleotide phosphate, **nicotinamide-beta-riboside**↑,NOS2, NTSR1, PARP1, phosphocreatine, PPARGC1A, **propionyl-carnitine**↑,PTGDR, RPTOR, **sarcosine**↓, **trigonelline**↓	18	15	
	RM10 T0	**1-aminocyclopropane-1-carboxylic acid**↓, **aceturic acid**↓, **adenine**↑, AGT, **beta-lactose**↑, cholesteryl oleate, **D-gluconic acid**↑, **D-ribose-5-phosphate**↑, **dihydrouracil**↑, ethanol, Glycine receptor, **glycocholic acid**↑, **glycocyamine**↑, **glycoursodeoxycholic acid**↑, GRIN1, GRIN3A, GRIN3B, IL1B, IL37, **kynurenic acid**↓, LILRA3, MMP9, **N'-methyl-2-pyridone-5-carboxamide**↓, **N-acetylneuraminic acid**↓, **nicotinamide-beta-riboside**↓, nitrate, PARP1, phosphocreatine, PPARGC1A, ,PTGDR, RPTOR, **sarcosine**↓, SL5A2, SLCO1B1, **taurine**↓, **trigonelline**↓	33	17	
	RM10 T4	**1-aminocyclopropane-1-carboxylic acid**↑, **aceturic acid**↓, **adenine**↓, AGT, **beta-lactose**↑, cholesteryl oleate, **D-gluconic acid**↑, **D-ribose-5-phosphate**↓, **dihydrouracil**↑, ethanol, Glycine receptor, **glycocholic acid**↑, **glycocyamine**↓, **glycoursodeoxycholic acid**↑, GRIN1, GRIN3A, GRIN3B, IL1B, IL37, **kynurenic acid**↑, **L-glutamine**↓, LILRA3, MMP9, **N'-methyl-2-pyridone-5-carboxamide**↓, **N-acetylneuraminic acid**↑, **nicotinamide-beta-riboside**↓, nitrate, PARP1, phosphocreatine, PPARGC1A, ,PTGDR, RPTOR, **sarcosine**↓, SL5A2, SLCO1B1, **taurine**↓, **trigonelline**↑	36	18	

	RM30 T0	**1-aminocyclopropane-1-carboxylic acid**↓,8-epi-prostaglandin F2alpha, **aceturic acid**↓, **adenine**↑, AGT, **beta-lactose**↑, cholesteryl oleate, **D-gluconic acid**↑, **D-ribose-5-phosphate**↑, **dihydrouracil**↑, ethanol, Glycine receptor, **glycocholic acid**↑, **glycocyamine**↓, **glycoursodeoxycholic acid**↓, GRIN1, GRIN3A, GRIN3B, IL1B, IL37, **kynurenic acid**↓, LILRA3, lipoxygenase, MMP9, **N'-methyl-2-pyridone-5-carboxamide**↓, **N-acetylneuraminic acid**↓, **nicotinamide-beta-riboside**↓, nitrate, PARP1, phosphocreatine, PPARGC1A, ,PTGDR, RPTOR, **sarcosine**↓, SL5A2, SLCO1B1, **taurine**↓, **trigonelline**↓	33	17
	RM30 T4	**1-aminocyclopropane-1-carboxylic acid**↓, **aceturic acid**↓, **adenine**↓, AGT, **beta-lactose**↑, cholesteryl oleate, **D-gluconic acid**↑, **D-ribose-5-phosphate**↓, **dihydrouracil**↑, ethanol, Glycine receptor, **glycocholic acid**↑, **glycocyamine**↑, **glycoursodeoxycholic acid**↑, GRIN1, GRIN3A, GRIN3B, IL1B, IL37, **kynurenic acid**↓, **L-glutamine**↓, LILRA3, MMP9, **N'-methyl-2-pyridone-5-carboxamide**↓, **N-acetylneuraminic acid**↑, **nicotinamide-beta-riboside**↓, nitrate, PARP1, phosphocreatine, PPARGC1A, ,PTGDR, RPTOR, **sarcosine**↓, SL5A2, SLCO1B1, **trigonelline**↑	33	17
	DHM T4	**1-aminocyclopropane-1-carboxylic acid**↑,6-keto-prostaglandin F1 alpha, **aceturic acid**↑, **adenine**↓, AGT, **beta-lactose**↑, CYP4A, **D-gluconic acid**↑, **D-ribose-5-phosphate**↓, **dihydrouracil**↓, ethanol, Glycine receptor, **glycocholic acid**↑, **glycocyamine**↑, **glycoursodeoxycholic acid**↑, GRIN1, GRIN3A, GRIN3B, IL1B, IL37, **kynurenic acid**↑, LILRA3, lipoxygenase, MMP9, **N'-methyl-2-pyridone-5-carboxamide**↓, **N-acetylneuraminic acid**↑, **nicotinamide-beta-riboside**↑, nitrate, PARP1, phosphocreatine, PPARGC1A, ,PTGDR, RPTOR, **sarcosine**↓, SL5A2, SLCO1B1, **taurine**↓, **trigonelline**↑	33	17

Molecules are grouped by Networks. The molecules identified are highlighted in bold, and the arrow indicates increase (up arrow) or decrease (down arrow) in their concentration in each data group compared to DHM at T0. Focus molecules represent the number of compounds identified in the dataset that are involved in the Network.

Table S7. Pathway Impact.

Pathway Name	Total	Hits	Raw p	-log(p)	Impact
Aminoacyl-tRNA biosynthesis *	48	20	5.09E-10	21.399	0.17
Arginine biosynthesis *	14	6	0.000738	7.2119	0.42
Pantothenate and CoA biosynthesis *	19	7	0.000772	7.167	0.06
Glycine, serine and threonine metabolism *	33	9	0.001635	6.4164	0.61
Alanine, aspartate and glutamate metabolism *	28	8	0.002158	6.1388	0.62
Valine, leucine and isoleucine biosynthesis *	8	4	0.003174	5.7528	0.00
Nicotinate and nicotinamide metabolism *	15	5	0.00751	4.8915	0.22
beta-Alanine metabolism *	21	6	0.007885	4.8429	0.22
Tryptophan metabolism *	41	9	0.007995	4.829	0.50

Histidine metabolism *	16	5	0.010155	4.5898	0.31
Pyrimidine metabolism *	39	8	0.018395	3.9957	0.26
Glyoxylate and dicarboxylate metabolism *	32	7	0.01922	3.9518	0.26
Ascorbate and aldarate metabolism *	8	3	0.0277	3.5863	0.50
Taurine and hypotaurine metabolism *	8	3	0.0277	3.5863	0.71
Phenylalanine, tyrosine and tryptophan biosynthesis *	4	2	0.041893	3.1726	1.00
Arginine and proline metabolism *	38	7	0.045969	3.0798	0.29
Phenylalanine metabolism	10	3	0.052015	2.9562	0.36
Nitrogen metabolism	6	2	0.093037	2.3748	0.00
D-Glutamine and D-glutamate metabolism	6	2	0.093037	2.3748	0.50
Glycerophospholipid metabolism	36	6	0.093815	2.3664	0.21
Purine metabolism	65	9	0.11688	2.1466	0.17
Pentose phosphate pathway	22	4	0.12519	2.0779	0.35
Butanoate metabolism	15	3	0.1426	1.9477	0.03
Cysteine and methionine metabolism	33	5	0.16393	1.8083	0.24
Glycerolipid metabolism	16	3	0.16466	1.8039	0.14
Neomycin, kanamycin and gentamicin biosynthesis	2	1	0.17019	1.7708	0.00
Lysine degradation	25	4	0.17682	1.7326	0.00
Vitamin B6 metabolism	9	2	0.18762	1.6733	0.49
Galactose metabolism	27	4	0.21465	1.5388	0.15
Glutathione metabolism	28	4	0.23435	1.4509	0.12
Citrate cycle (TCA cycle)	20	3	0.26062	1.3447	0.17
D-Arginine and D-ornithine metabolism	4	1	0.31159	1.1661	0.00
Linoleic acid metabolism	5	1	0.37304	0.98607	1.00
Thiamine metabolism	7	1	0.48007	0.73382	0.00
Pentose and glucuronate interconversions	18	2	0.48598	0.72158	0.13
Porphyrin and chlorophyll metabolism	30	3	0.50844	0.67641	0.03
Inositol phosphate metabolism	30	3	0.50844	0.67641	0.13
Fructose and mannose metabolism	20	2	0.54373	0.6093	0.04
Ubiquinone and other terpenoid-quinone biosynthesis	9	1	0.56894	0.56398	0.00
Sphingolipid metabolism	21	2	0.57088	0.56057	0.01
Primary bile acid biosynthesis	46	4	0.59776	0.51456	0.02
Caffeine metabolism	10	1	0.60754	0.49833	0.00
Biotin metabolism	10	1	0.60754	0.49833	0.00
Valine, leucine and isoleucine degradation	40	3	0.70701	0.3467	0.00
Starch and sucrose metabolism	18	1	0.81515	0.20439	0.42
Biosynthesis of unsaturated fatty acids	36	2	0.8458	0.16747	0.00

Selenocompound metabolism	20	1	0.84696	0.1661	0.00
Ether lipid metabolism	20	1	0.84696	0.1661	0.00
Amino sugar and nucleotide sugar metabolism	37	2	0.85673	0.15463	0.05
Pyruvate metabolism	22	1	0.87333	0.13544	0.08
Propanoate metabolism	23	1	0.88477	0.12243	0.00
Glycolysis / Gluconeogenesis	26	1	0.91329	0.090703	0.00
Phosphatidylinositol signaling system	28	1	0.92829	0.074415	0.04
Arachidonic acid metabolism	36	1	0.96654	0.034036	0.31
Tyrosine metabolism	42	1	0.98116	0.01902	0.14

The impact in the metabolic pathways was analyzed using MetaboAnalysis 4.0 software. Pathways with bold asterisk (*) are significantly impacted.
